# Vine‐Inspired Soft Robotics: Materials, Actuations, and Emerging Applications

**DOI:** 10.1002/advs.78022

**Published:** 2026-09-29

**Authors:** Jiahao Wu, Xingyun Liu, Fangzhou Wang, Huili Ren, Chaojun Zhang, Ziyu Ren, Zunqi Liu, Jing Li

**Affiliations:** ^1^ School of Chemistry Beihang University Beijing China; ^2^ Chemistry and Chemical Engineering College Xinjiang Agricultural University Urumqi Xinjiang China; ^3^ School of Mechanical Engineering and Automation Beihang University Beijing China; ^4^ Department of Cardiovascular Surgery & School of Medicine, Zhongda Hospital Southeast University Nanjing Jiangsu China

**Keywords:** bioinspired design, multimodal actuation, soft robotics, tip growth, vine robots

## Abstract

Inspired by the tip‐growth behavior of natural vines, vine‐inspired robots have emerged as a distinctive paradigm in soft robotics, enabling adaptive extension and navigation in confined, fragile, and highly unstructured environments. Unlike conventional robots that rely on whole‐body locomotion, vine‐inspired robots extend by generating or deploying their body from the tip, making performance strongly dependent on material properties, interfaces, and processing strategies. This review provides a comprehensive overview of recent advances from a materials perspective. We first examine material strategies for fluid‐driven eversion and phase‐transition‐ or deposition‐enabled growth, linking material chemistry, geometry, interfaces, and phase behavior to growth performance. We then discuss how material–structure design supports steering, retraction, multifunctional tip design, variable stiffness, climbing and attachment, and branching, while evaluating their performance trade‐offs and technological maturity. Representative applications in medical diagnosis and intervention, navigation and exploration, transport and operational tasks, and deployable and reconfigurable structures are highlighted. Finally, we discuss key challenges in hierarchical material integration, scalable manufacturability, long‐term reliability, and morphology‐aware sensing and control, together with opportunities enabled by learning‐based approaches and embodied intelligence. By connecting materials, growth mechanisms, functional capabilities, and application requirements, this review provides a unified framework for next‐generation adaptive growing robots.

## Graphical abstract

Vine‐inspired robots grow from the tip, enabling navigation through confined spaces with minimal disturbance. This Review summarizes material and actuation strategies for vine‐inspired growth, key functions including steering, retraction, attachment, branching, and variable stiffness, and emerging applications spanning medical intervention, confined‐space navigation, internal transport, and deployable structures.

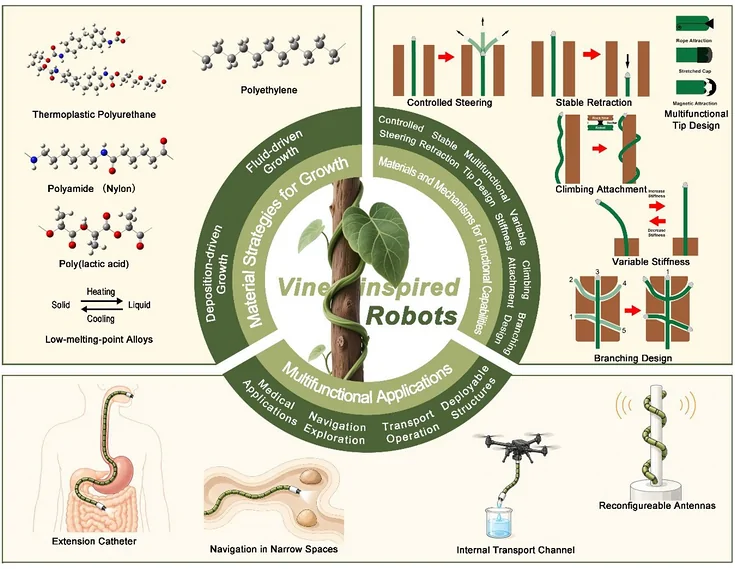

## 1 Introduction

Over the course of evolution, living organisms have developed two distinct strategies for interacting with their environments and acquiring resources: locomotion and growth. Locomotion, typified by animals, relies on frictional interactions between the body and the environment to generate propulsion and whole‐body displacement. Growth, exemplified by plants, enables spatial expansion through localized material addition and deformation. Among plant systems, vines are a compelling source of inspiration because they extend, explore, and adapt through tip growth [1, 2]. The previously formed body remains largely stationary while new length is generated near the apex. This strategy limits friction along the trailing body and allows vines to enter dense vegetation, narrow crevices, and subsurface spaces.

Biological tip growth is governed by coordinated processes across multiple scales. According to the Lockhart description of cell elongation [3], internal turgor pressure drives expansion when the stress in the cell wall exceeds a yield threshold. The location and rate of growth are further regulated by water transport, cell‐wall remodeling, biochemical signaling, and environmental sensing. Vines can redirect growth in response to light and contact through phototropism [4] and thigmotropism [5], while tissue maturation and lignification increase structural stability after elongation [6, 7]. Their adaptability therefore cannot be attributed to material properties alone. It arises from the coupling of sensing, transport, growth regulation, and evolving material mechanics. Once formed, the grown body also supports load‐bearing, transport, attachment, and interaction with the environment.

Inspired by this biological strategy, vine robots generate or deploy new body length near the tip while limiting translation of the previously deployed section. Two principal growth mechanisms have been developed. Fluid‐driven robots use pressure to evert thin‐film bodies and reproduce the kinematic outcome of apical extension [8]. This mechanism is functionally similar to vine growth, although it does not reproduce the biological process of adding new cellular material. Phase‐transition and material‐deposition robots instead transport a feedstock to the tip and locally solidify it through phase transition or melt processing [9, 10]. This approach more closely resembles the localized addition and stabilization of material in plant growth. Both strategies reduce frictional disturbance along the deployed body and support navigation in confined or sensitive environments [11]. Representative applications include minimally invasive intervention, archaeological and infrastructure inspection, environmental exploration, and remote material transport [12, 13, 14, 15].

Recent advances in soft materials provide additional routes for embedding sensing, actuation, and multifunctionality into compliant robotic systems. Three‐dimensional‐printed PEDOT: PSS combines mechanical flexibility with mixed ionic and electronic conductivity, making it attractive for soft sensors, electrodes, and actuators. Its performance and integration depend strongly on ink rheology, post‐processing, and interface design [16]. Multimaterial hydrogels can combine compliant, rigid, and functional components within complex three‐dimensional architectures. Their low‐density polymer networks, however, make shape retention and mechanically robust interfaces difficult without specialized processing [17, 18]. Recent biphasic dielectric elastomers demonstrate high electromechanical sensitivity and strong actuation output under reduced driving fields. Their performance still depends on the coupled optimization of modulus, dielectric constant, breakdown strength, compliant electrodes, and cyclic stability [19]. These material systems have greatly expanded the functional capabilities of flexible robotics, but they do not necessarily meet the structural and processing requirements of growing robotic bodies.

Vine robots impose a distinctive combination of structural and processing demands. A fluid‐driven body must fold into a compact volume, pass through the eversion front with low resistance, retain pressure along long seams, tolerate tearing and abrasion, and form a load‐bearing shell after inflation. Thin polyethylene (PE) films, thermoplastic polyurethane (TPU), and coated nylon fabrics meet these requirements through low areal mass, film or textile architectures, thermoplastic sealing, and established scalable processing. Phase‐transition and deposition systems require a different balance. Low‐melting‐point alloys provide reversible stiffness regulation and shape locking, whereas poly(lactic acid) (PLA) and polycaprolactone (PCL) combine melt flow, interlayer bonding, and post‐growth structural support. These materials generally provide fewer intrinsic sensing or actuation functions than hydrogels, conducting polymers, or electroactive elastomers. Their pressure integrity, dimensional stability, joinability, and process reliability nevertheless remain advantageous for long, self‐supporting, or pressure‐bearing growing bodies. Emerging soft materials are therefore particularly valuable as functional skins, local actuators, sensors, interfaces, or tip modules that complement the primary structural material.

Comprehensive reviews have categorized tip‐growing robots according to their working principles, designs, fabrication processes, actuation and steering methods, mechanics, control, and applications [11, 20, 21]. The present review complements these system‐level perspectives by establishing a critical map between material properties and robotic functions (Figure 1). Section 2 examines how material chemistry, processing history, geometry, interfaces, and phase behavior govern fluid‐driven eversion, phase transition, and deposition growth. Section 3 discusses material contributions to steering, retraction, multifunctional tip design, variable stiffness, climbing and attachment, and branching. The level of experimental validation is considered alongside the proposed mechanisms. Section 4 relates material selection to application‐specific requirements in medicine, exploration, transport, and deployable or reconfigurable structures. Particular attention is given to manufacturability, interfacial reliability, cyclic durability, and environmental stability. By linking materials, mechanisms, functions, and application constraints, this review provides a materials‐based framework for the design and evaluation of vine‐inspired soft robots (Figure 2).

**FIGURE 1:**
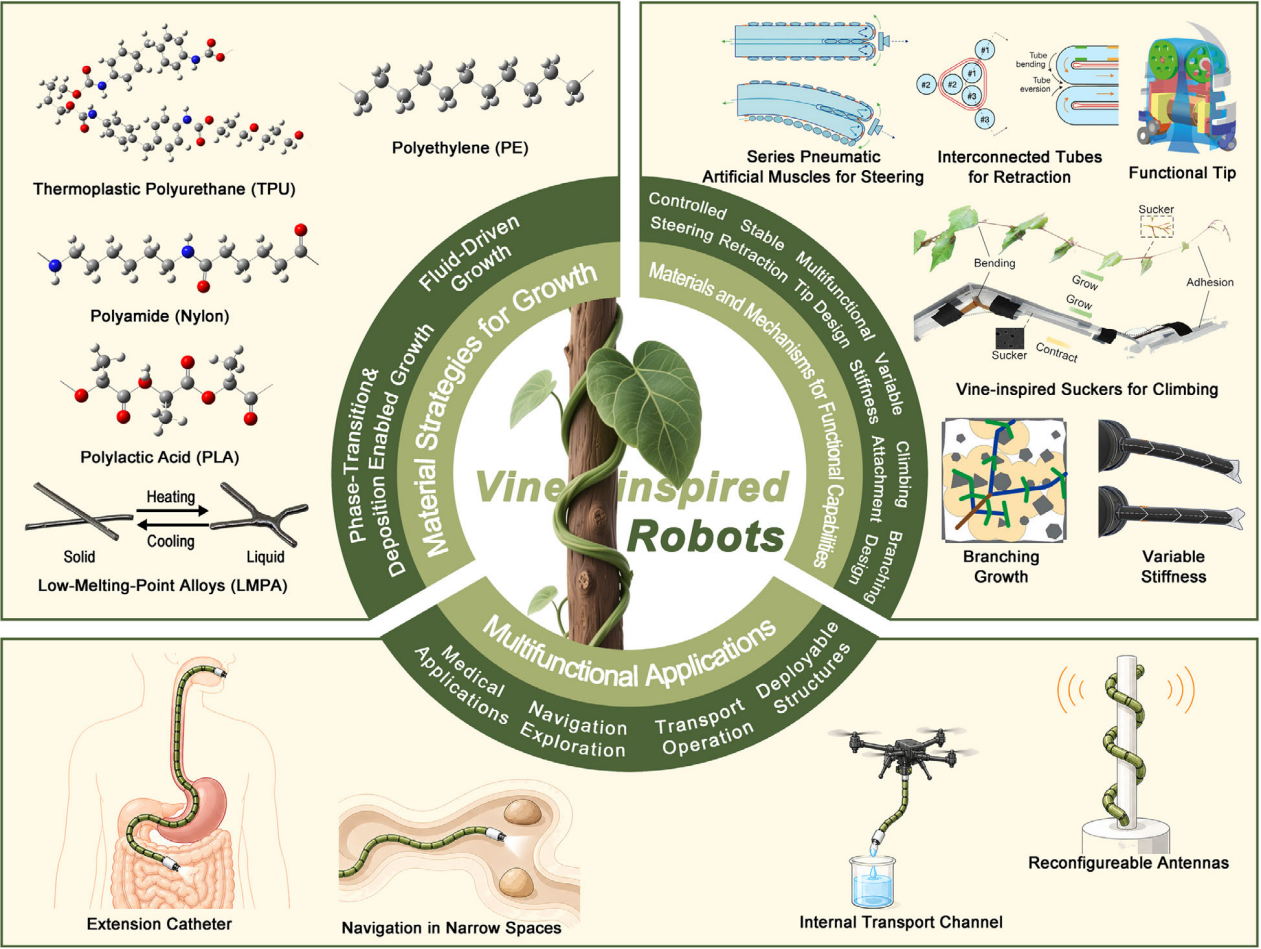
Overview of vine‐inspired robots, linking growth materials, functional capabilities, and application scenarios. The left panel summarizes structural materials for fluid‐driven and phase‐transition and deposition growth; the right panel highlights steering, retraction, multifunctional tips, climbing and attachment, branching, and variable stiffness; the lower panel shows representative applications in medical intervention, confined‐space navigation, internal transport, and deployable structures. Low‐melting‐point Alloys: Adapted with permission [22]. Copyright 2021, The Authors, published by Wiley‐VCH GmbH. Steering module: Adapted with permission [23]. Copyright 2019, Mary Ann Liebert, Inc. Retraction module: Adapted with permission [24]. Copyright 2024, IEEE. Functional tip: Adapted with permission [25]. Copyright 2020, IEEE. Vine‐inspired suckers: Reproduced with permission [26]. Copyright 2025, AAAS. Branching growth: Reproduced under the terms of the CC BY 4.0 license [27]. Copyright 2022, The Authors, published by Frontiers. Variable stiffness: Adapted with permission [28]. Copyright 2024, Mary Ann Liebert, Inc. Other schematics were created by the authors. sPAMs, series pneumatic artificial muscles.

**FIGURE 2:**
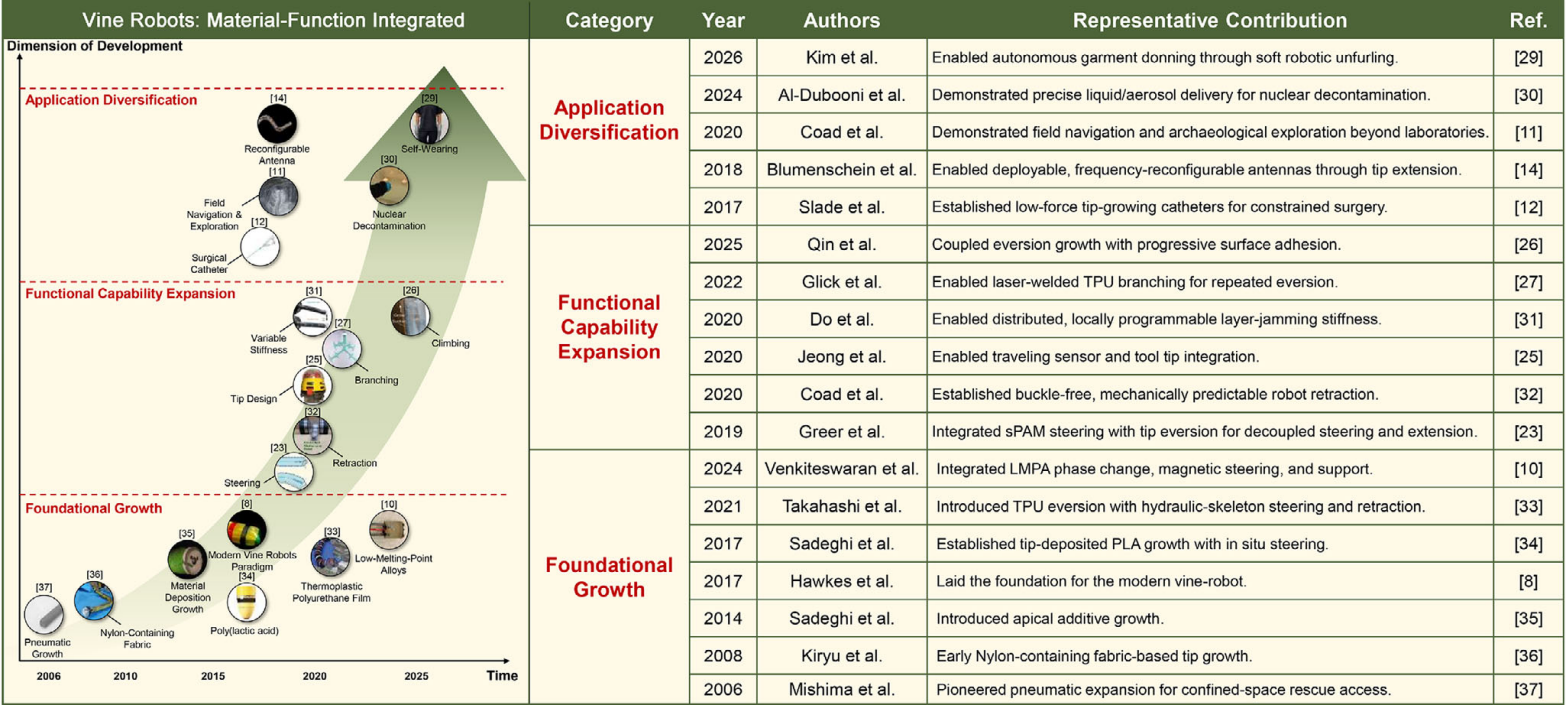
Material–function evolution of vine‐inspired robots. The left panel traces representative milestones from foundational growth mechanisms to functional capability expansion and application diversification. The right panel summarizes the publication year, authors, representative contribution, and corresponding reference for each milestone. Milestones were selected based on peer‐reviewed hardware demonstrations that established (i) a distinct growth/body‐formation or material‐processing route, (ii) a material‐, interface‐, or structure‐enabled functional capability integrated into a growing robot, or (iii) an application‐oriented system demonstration. Each study is assigned to its primary contribution. The categories denote thematic developmental dimensions rather than strictly sequential stages, and vertical position does not indicate a quantitative ranking of performance or technological maturity. Representative images, in the order shown in the table: Self‐wearing: Reproduced with permission [29]. Copyright 2026, IEEE. Nuclear Decontamination: Reproduced with permission [30]. Copyright 2024, IEEE. Field navigation and exploration: Reproduced with permission [11]. Copyright 2020, IEEE. Reconfigurable antenna: Reproduced with permission [14]. Copyright 2018, IEEE. Surgical catheter: Reproduced with permission [12]. Copyright 2017, IEEE. Climbing robot: Reproduced with permission [26]. Copyright 2025, AAAS. Branching robot: Reproduced under the terms of the CC BY 4.0 license [27]. Copyright 2022, The Authors. Published by Frontiers. Variable stiffness: Reproduced with permission [31]. Copyright 2020, IEEE. Tip‐mounted sensor and tool integration: Reproduced with permission [25]. Copyright 2020, IEEE. Retraction: Reproduced with permission [32]. Copyright 2020, IEEE. Steering: Reproduced with permission [23]. Copyright 2019, Mary Ann Liebert, Inc. LMPA‐based growth: Reproduced with permission [10]. Copyright 2024, IEEE. Hydraulic‐skeleton steering: Reproduced with permission [33]. Copyright 2021, IEEE. PLA deposition growth: Reproduced with permission [34]. Copyright 2017, Mary Ann Liebert, Inc. Modern Vine Robots Paradigm: Reproduced with permission [8]. Copyright 2017, The Authors; exclusive licensee AAAS. Material deposition growth: Reproduced under the terms of the CC BY 4.0 license [35]. Copyright 2014, The Authors. Published by PLOS. Nylon‐containing fabric growth: Reproduced with permission [36]. Copyright 2008, The Japan Fluid Power System Society. Pneumatic Growth: Reproduced with permission [37]. Copyright 2006, SAGE Publications.

## 2 Material Strategies for Vine‐Inspired Robots

The growth behavior of vine robots is determined not only by their macroscopic architecture but also by how constituent materials deform, flow, bond, and change state during extension. For materials analysis, this section treats fluid‐driven eversion separately from phase‐transition and deposition‐enabled strategies, while recognizing that some low‐melting‐point alloy (LMPA) systems combine phase change with fluidic eversion. Fluid‐driven robots require thin, foldable, and pressure‐retaining bodies that can pass through the eversion front with low resistance while maintaining sufficient strength, seam integrity, and damage tolerance. Polyethylene films, thermoplastic polyurethane, and coated nylon fabrics exhibit different balances between flexibility and durability. Phase‐transition systems based on low‐melting‐point alloys instead use localized solid–liquid switching to create a movable compliant region and restore load‐bearing stiffness after cooling. Thermoplastics such as PLA and PCL generate new body structure through localized melting, deposition, solidification, and interlayer bonding. These strategies therefore differ in whether the robot body is prefabricated, mechanically reconfigured, or constructed in situ. This section examines how material chemistry, processing history, geometry, interfaces, and thermal behavior collectively govern growth efficiency, mechanical performance, manufacturability, and long‐term reliability.

### 2.1 Material Requirements for Fluid‐Driven Eversion

#### 2.1.1 Polyethylene

Polyethylene (PE) is widely used in fluid‐driven vine robots because it can be processed into lightweight, flexible, and heat‐sealable films. It is a semicrystalline polyolefin composed of nonpolar, saturated ─CH_2_─CH_2_─ chains [40]. Commercial PE families differ in chain branching, density, and molecular weight. They include low‐density polyethylene (LDPE), linear low‐density polyethylene (LLDPE), high‐density polyethylene (HDPE), and ultra‐high‐molecular‐weight polyethylene (UHMWPE) (Figure 3A) [41]. Among these grades, LDPE is the most frequently used in pressure‐driven vine robots. LDPE contains both short‐ and long‐chain branches. These branches disrupt regular chain packing and reduce crystallinity relative to more linear PE grades [40]. Consequently, LDPE generally exhibits low density and stiffness, together with high ductility and foldability. In thin‐film form, it can be folded or wound into a compact storage volume. It can also pass through the highly curved eversion front with relatively low resistance.

**FIGURE 3:**
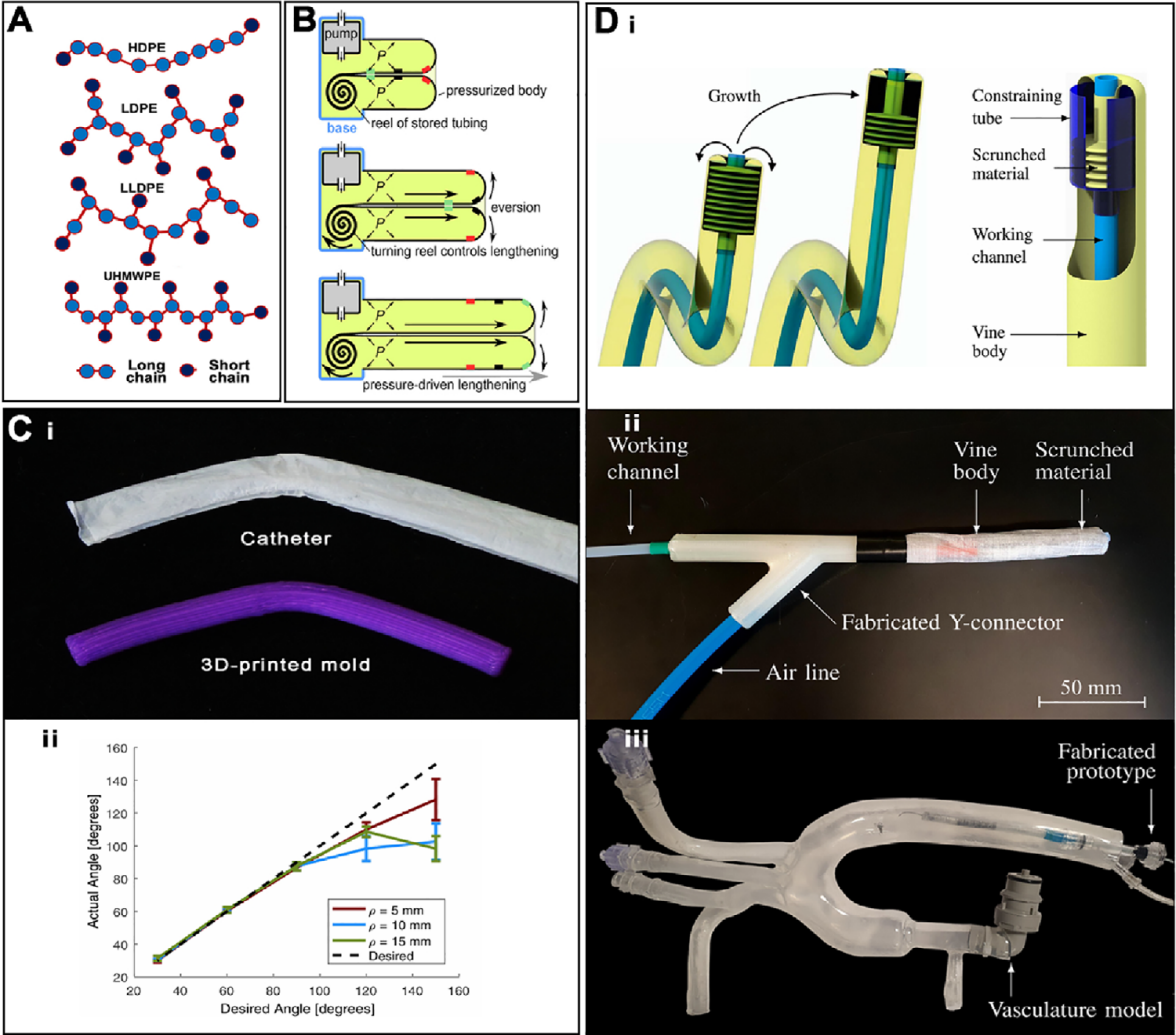
Polyethylene structures and representative fluid‐driven vine robots. (A) Chain architectures of HDPE, LDPE, LLDPE, and UHMWPE. (B) Reel‐controlled pressure‐driven tip eversion. (C) (i) Pre‐formed LDPE catheter and 3D‐printed mold and (ii) pre‐formed bend angles at various radii of curvature and with standard deviation bars. (D) (i) Material‐scrunching miniature vine robot structure, (ii) vine robot with scrunched material and a working channel, (iii) 5.2 mm diameter everting vine robot inside an aorta phantom. (A) Adapted under the terms of the Creative Commons Attribution‐Non Commercial 4.0 International License [38]. Copyright 2026, The Authors, published by Wiley Periodicals LLC on behalf of the Society of Plastics Engineers. (B) Reproduced with permission [8]. Copyright 2017, AAAS. (C) Reproduced with permission [12]. Copyright 2017, IEEE. (D) Reproduced with permission [39]. Copyright 2024, IEEE. HDPE, high‐density polyethylene; LDPE, low‐density polyethylene; LLDPE, linear low‐density polyethylene; PE, polyethylene; UHMWPE, ultra‐high‐molecular‐weight polyethylene.

The properties of PE films depend strongly on resin grade, thickness, additives, and manufacturing process. Representative commercial LDPE film grades have densities of approximately 0.92 g cm^−^^3^, 1% secant moduli in the low hundreds of megapascals, tensile strengths at break of approximately 18–29 MPa, and high elongations at break [42, 43, 44]. These values should not be regarded as fixed intrinsic constants. Blown‐film processing introduces molecular and crystalline orientation, resulting in different tensile, tear, and fracture responses in the machine and transverse directions [45, 46]. Film grade and processing conditions also affect optical clarity, surface friction, and heat‐sealing behavior. Therefore, identifying a robot body simply as “LDPE” is insufficient. Meaningful comparison requires the reporting of film grade, wall thickness, processing method, film orientation, and joining technique.

The suitability of LDPE for pressure‐driven growth arises from three coupled material functions. First, its relatively low modulus and high foldability reduce the energy required to bend the film at the eversion front. Second, its low areal mass allows long robot bodies to be stored compactly without translating the previously deployed section. Third, its thermoplastic nature enables the fabrication of heat‐sealed tubular bodies, pneumatic chambers, and other integrated structures. Together, these properties support smooth tip eversion without requiring large elastic strains throughout the film.

As a first‐order approximation, the effect of film geometry can be described by Equation (1) using the bending rigidity of an isotropic plate [47]:

(1) $$D=\frac{Et^{3}}{12\left(1\;-\;\nu^{2}\right)}\;$$

where *D* is the bending rigidity, *E*, *t*, and ν are the elastic modulus, film thickness, and Poisson's ratio, respectively. Although processed PE films may be anisotropic, Equation (1) is used here only as a qualitative approximation to illustrate the dominant effect of thickness. Because bending rigidity scales with the third power of thickness, a small reduction in wall thickness can markedly decrease the bending‐related resistance at the eversion front. Ultrathin LDPE films are therefore attractive for low‐force growth and miniaturized systems. After pressurization, the initially flat film forms a cylindrical shell with substantially greater geometric stiffness. The same material can thus remain compliant during storage while forming a load‐bearing body after deployment.

This advantage is accompanied by a fundamental pressure–durability trade‐off. For a thin cylindrical body, the nominal circumferential stress can be approximated by Equation (2) [48]:

(2) $$\sigma_{\theta }=\frac{pr}{t}\;$$

where *p*, *r*, and *t* are the internal pressure, robot radius, and wall thickness, respectively. At a fixed pressure and radius, reducing the wall thickness increases the membrane stress. A thinner film therefore lowers the bending‐related component of the eversion pressure but does not improve the pressure‐bearing capacity of the body. Instead, it narrows the safe operating range and increases sensitivity to scratches, pinholes, puncture, tearing, and local heat‐seal defects. Material and geometric design must consequently balance low eversion resistance against burst pressure and damage tolerance.

Pressure retention is another essential requirement for fluid‐driven growth. It is a system‐level property governed by the film, sealed seams, and end connections. LDPE has finite gas permeability and should therefore be described as sufficiently gas‐retentive over the intended operating period rather than intrinsically airtight [49]. Seam reliability depends on sealing temperature, dwell time, applied pressure, film orientation, and interfacial cleanliness [50]. Insufficient heating can cause incomplete fusion and leakage. Excessive heating may thin, distort, or weaken the joint. These processing variables directly affect burst pressure, stable eversion, and batch‐to‐batch reproducibility. Nevertheless, LDPE remains attractive because it is widely available, easy to cut, and compatible with rapid and scalable heat‐sealing processes. These advantages make it one of the most cost‐effective materials for laboratory‐scale vine robots and disposable prototypes [51].

The historical development of pressure‐driven tip‐growing systems illustrates how thin PE films enabled the transition from expandable devices to modern vine robots. Before the term “vine robot” was introduced, Mishima et al. [37] developed the pneumatically driven Slime Scope for rescue operations in confined spaces. This device can be regarded as an early precursor of later fluid‐driven growing robots. The broad scalability of thin PE films was subsequently demonstrated by Hawkes et al. [8]. Their pressure‐driven robots used PE bodies approximately 50–80 µm thick and achieved tip growth through material eversion (Figure 3B). A pneumatic system controlled the internal pressure, while a rotating spool regulated the release of stored body material. Robots were demonstrated with diameters ranging from 1.8 mm to 36 cm. One system extended from an initial length of 28 cm to 72 m, and peak velocities of approximately 10 m s^−1^ were reported over short distances. These results demonstrate the compact storage, low moving mass, and scalability enabled by thin‐film bodies. However, the high growth velocity cannot be attributed solely to the tensile properties of LDPE. It resulted from the combined effects of film thickness, robot diameter, internal pressure, pneumatic flow capacity, and the material‐release mechanism.

At smaller scales, Slade et al. [12] fabricated pre‐formed tip‐extending catheters using 13 µm LDPE film. The authors selected the thin film to minimize bending stiffness and eversion resistance, which facilitated apical extension and accurate reproduction of pre‐formed bend angles (Figure 3C). However, miniaturization introduces additional constraints. As the robot cross‐section is reduced, internal friction becomes increasingly significant relative to the pressure‐generated driving force, leading to substantially higher growth pressures. Girerd et al. [39] addressed this limitation by modifying the material‐storage and release architecture. They used LDPE films to fabricate a robot with a diameter of 2.3 mm (Figure 3D). In conventional systems, the material is stored at the base and pulled from a spool during growth. In contrast, Girerd et al. compressed the film into layers and stored it near the front of the robot, allowing direct release during extension. This configuration design decoupled the driving pressure from robot length and prevented friction from accumulating as the robot grew. It also enabled the integration of a continuous working channel within a highly miniaturized body. This study shows that robot performance is governed not by film compliance alone, but by its coupling with storage architecture, contact interfaces, and internal friction.

Long‐term reliability is affected by repeated folding, bending, pressurization, eversion, and environmental contact. LDPE is viscoelastic, and prolonged storage or sustained loading can cause creep, stress relaxation, wrinkling, and residual curvature [52]. These changes may distort the eversion front, increase the pressure required for growth, and produce asymmetric deployment. Monotonic tensile measurements alone cannot predict such behavior. PE films with similar tensile strengths may exhibit different fatigue lives because of differences in molecular architecture, processing‐induced orientation, thickness, and seam quality [53]. Reusable vine robots should therefore be assessed through cyclic pressurization and eversion tests rather than only through single‐cycle tensile or burst measurements. Environmental aging introduces further limitations. Ultraviolet radiation, oxygen, and elevated temperatures can oxidize PE, alter its crystalline structure, increase stiffness, and reduce ductility [54, 55]. For a vine robot, aging‐induced stiffening increases resistance at the eversion front, whereas embrittlement reduces tolerance to folds, scratches, and repeated deployment. UV‐stabilized grades, multilayer films, protective coatings, and fiber reinforcement may improve durability. However, these strategies can also increase wall thickness, bending rigidity, manufacturing complexity, and the risk of interfacial failure. For long‐term operation in natural or otherwise harsh environments, unmodified PE may therefore be insufficient, and the stability of the complete material system must be considered.

Overall, PE illustrates how molecular architecture, processing history, geometry, and joining quality collectively determine robotic performance. Chain branching and crystallinity affect stiffness, flexibility, and toughness. Film thickness governs both bending resistance and membrane stress, while heat‐seal quality determines pressure retention. Viscoelasticity, fatigue, and environmental aging further control long‐term repeatability. LDPE is therefore particularly suitable for low‐cost prototypes, rapid or long‐distance eversion, and applications involving relatively benign contact conditions.

#### 2.1.2 Thermoplastic Polyurethane

Compared with PE, TPU is attractive when a vine robot requires a higher burst‐pressure margin, improved tear resistance, or multichamber and branched welded architectures. Polyurethane refers to a broad family of polymers containing urethane linkages (Figure 4A). It includes permanently crosslinked thermosets and melt‐processable thermoplastics. Crosslinked PU generally cannot be melt‐reprocessed after curing. By contrast, TPU consists predominantly of linear segmented chains connected through reversible physical associations. It can therefore soften upon heating and recover an elastomeric network after cooling [58]. A typical TPU is synthesized from a long‐chain polyol, a diisocyanate, and a short‐chain extender. The polyol‐rich soft segments provide chain mobility and extensibility, whereas the urethane‐rich hard segments form polar, hydrogen‐bonded associations. Thermodynamic incompatibility between these segments produces nanoscale phase separation. The hard domains serve as physical crosslinks and reinforcing regions, while the soft phase forms a deformable matrix [59]. This microphase‐separated morphology provides a tunable balance between mechanical strength and flexibility. Increasing the hard‐segment content or domain order generally raises the modulus, strength, and thermal resistance. However, it can also reduce extensibility and increase resistance at the eversion front. A larger soft‐segment fraction improves flexibility and foldability but may reduce dimensional stability and pressure‐bearing capacity.

**FIGURE 4:**
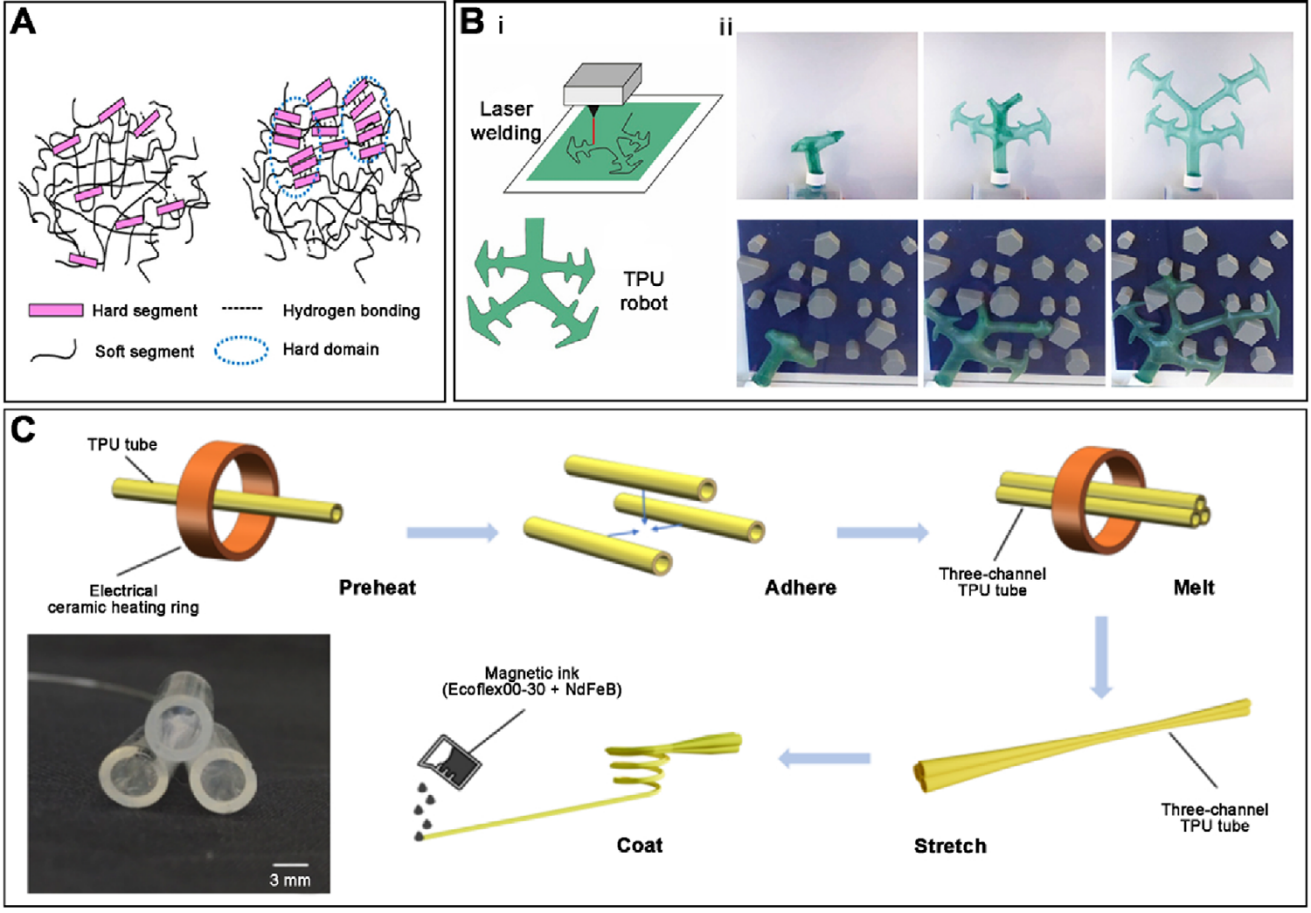
TPU morphology, fabrication, and robotic integration. (A) Segmented TPU microstructure with hydrogen‐bonded hard domains and a deformable soft phase. (B) (i) Laser‐welded branching body and (ii) sequential branch deployment. (C) Thermal drawing of a three‐channel TPU tube followed by a distal Ecoflex/NdFeB composite coating. (A) Adapted under the terms of the Creative Commons Attribution 4.0 International License [56]. Copyright 2022, The Authors, published by MDPI. (B) Reproduced under the terms of the CC BY 4.0 license [27]. Copyright 2022, The Authors, published by Frontiers. C) Reproduced with permission [57]. Copyright 2025, American Chemical Society. NdFeB, neodymium–iron–boron; TPU, thermoplastic polyurethane.

Representative TPU films can exhibit tensile strengths of approximately 30–70 MPa and elongations at break of 200%–900% [41]. Their Young's modulus can be adjusted over a broad range, approximately 10–500 MPa, through changes in segment chemistry, composition, and morphology. These values are strongly dependent on material grade, hardness, processing history, film thickness, and test method. They should therefore not be treated as fixed intrinsic properties. Compared with commonly used LDPE films, many TPU grades provide higher tensile strength and greater resistance to tearing and repeated deformation.

The balance between strength and flexibility directly affects pressure‐driven growth. TPU films can provide greater resistance to membrane tension, repeated folding, and obstacle contact than thin commodity PE films. Nevertheless, high tensile strength alone does not ensure efficient eversion. As discussed for PE, film bending rigidity increases approximately with *Et* ^3^, while nominal circumferential stress scales with *pr*/*t*. A thicker TPU wall increases the pressure margin and improves puncture resistance, but it also increases bending resistance, storage volume, and the pressure required to pass through the eversion front. In addition, TPU grades with a high modulus or hardness may resist folding even when their tensile strength is high. Material selection must therefore balance hardness, modulus, thickness, robot radius, surface friction, and seam stiffness. TPU should not be regarded as a universally superior replacement for PE.

An important advantage of TPU is its compatibility with thermoplastic fabrication. Heating weakens hydrogen bonding and disrupts associations between hard domains, allowing chain motion and viscous flow. Cooling restores these physical associations and rebuilds the elastomeric network [58, 59]. Depending on the grade and processing configuration, this reversible transition enables heat sealing, impulse sealing, laser welding, extrusion, reshaping, and thermal drawing. These processes can produce sealed pneumatic chambers and geometrically complex robot bodies without permanently curing the polymer [27].

The thermal behavior of TPU must nevertheless be described carefully. The glass‐transition temperature of the soft phase is commonly below room temperature and may lie approximately between −50°C and −30°C, depending on composition. This low transition temperature helps retain flexibility under moderately cold conditions. TPU grades also exhibit melting or softening transitions that are often above 150°C. These transitions provide a useful thermoplastic processing window, but they do not define the continuous service temperature of the material. Thermal drawing further connects TPU processability with robotic miniaturization. In this process, a macroscopic preform is heated into a controlled viscoelastic state and drawn under tension. The cross‐section is reduced while embedded channels and other structural features remain continuous. The final dimensions depend on the temperature profile, draw tension, preform feed rate, and take‐up speed. Controlled draw‐down can produce microscale, fiber‐like bodies with continuous internal channels. This capability is particularly useful for miniaturized vine robots that require simultaneous fluid transport, actuation, sensing, or therapeutic functions. The process also requires a stable thermal window because local variations in viscosity or tension can affect dimensional uniformity and channel integrity.

TPU is viscoelastic, and its mechanical response changes with strain rate, loading history, and cycle number. It can exhibit hysteresis, residual strain, creep, stress relaxation, and stress softening during early loading cycles [60]. In some formulations, conditioning can also reorganize the hard domains and alter the subsequent strain‐hardening response [61]. These effects may change the eversion pressure, seam strain, bending curvature, and trajectory repeatability. Monotonic tensile measurements are therefore insufficient for material screening. Cyclic pressurization, repeated folding and eversion, seam fatigue, creep, and stress relaxation should also be evaluated under representative operating conditions.

Environmental stability depends strongly on the soft‐segment chemistry. Polyester‐, polyether‐, and polycarbonate‐based TPUs exhibit different responses to moisture, oxidation, and biological environments. Polyester‐based TPUs are generally more susceptible to hydrolytic degradation. Suitable polyether‐based formulations often provide improved resistance to hydrolysis, whereas polycarbonate‐based TPUs can offer favorable long‐term mechanical and biological stability [62]. However, no single chemistry is optimal under all conditions. Ultraviolet radiation, heat, oxygen, moisture, and mechanical damage can change the molecular weight, hydrogen‐bonding network, and phase morphology of TPU [63]. These changes may cause embrittlement, softening, permanent deformation, or weakening of welded seams. Material chemistry should therefore be selected according to the intended environment and service duration.

Representative robots illustrate how these material characteristics translate into progressively more complex system functions. Takahashi et al. [33] reported an early TPU‐based eversion robot that combined liquid‐driven extension with an internal hollow hydraulic skeleton. The inserted mechanism enabled directional extension and retraction, demonstrating the compatibility of TPU membranes with independently operated steering structures. Glick et al. [27] subsequently used a commercial TPU film, Stretchlon 200 from Fibre Glast, and laser welding to fabricate branched robot bodies (Figure 4B). The resulting structures tolerated internal pressures of up to 70 kPa. The work demonstrates how TPU weldability can support sealed and mechanically robust branching architectures. Liu et al. [57] used TPU as the structural material for a multifunctional vine robot intended for in vivo therapy. A macroscopic TPU preform was thermally processed and drawn into a fiber‐like structure with a diameter of approximately 300 µm (Figure 4C). The process preserved continuous microscale features and enabled the integration of multiple functional channels within a compact body. This example highlights a capability that is difficult to achieve using conventional heat‐sealed PE films: the scalable fabrication of highly miniaturized, multifunctional structures with continuous internal geometries.

Overall, TPU offers more than higher tensile strength. Its segmented molecular architecture provides tunable stiffness, toughness, elasticity, and thermal processability. These properties support higher‐pressure bodies, welded branching structures, and multifunctional miniaturization. At the same time, TPU introduces grade‐dependent hysteresis, creep, processing sensitivity, and environmental aging. Its performance in vine robots is therefore governed by the coupling among segment chemistry, phase morphology, film geometry, joining quality, and loading history. TPU is suitable when mechanical durability, complex fabrication, or microscale functional integration are more important than the minimum possible bending resistance and material cost.

#### 2.1.3 Polyamide (Nylon)‐Based Coated Textiles

Nylon is the conventional name for a family of synthetic polyamides rather than a single polymer. The most common textile grades are polyamide 6 (PA6, nylon 6) and polyamide 66 (PA66, nylon 6,6) (Figure 5A). PA6 is generally produced from caprolactam, whereas PA66 is formed from hexamethylenediamine and adipic acid. Both are semicrystalline aliphatic polyamides containing regularly repeated amide groups. Interchain hydrogen bonding, crystalline domains, and drawing‐induced molecular orientation give nylon fibers high tensile strength, toughness, and abrasion resistance [69, 70]. Their properties nevertheless depend on polymer grade, molecular weight, crystallinity, drawing ratio, moisture content, and textile processing. Representative nylon materials exhibit tensile properties higher than those of many commodity PE films [41], but the behavior of a vine‐robot wall is governed by the complete textile architecture.

**FIGURE 5:**
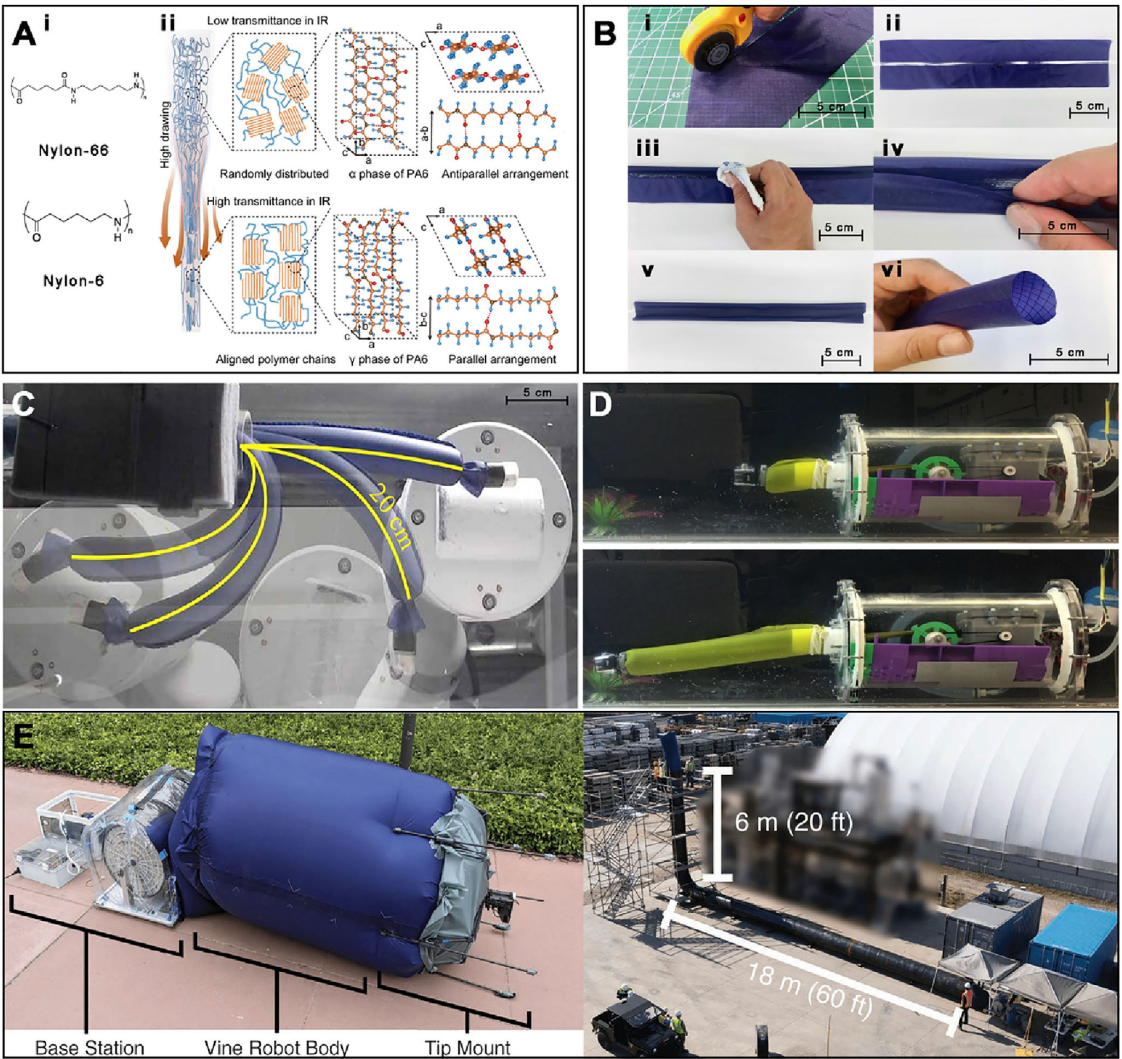
Polyamide chemistry and coated‐textile bodies for vine robots. (A) (i) Molecular structures of nylon 6,6 and nylon 6 and (ii) drawing‐induced chain alignment. (B) (i–vi) Fabrication and deformation of a bonded, coated ripstop nylon pneumatic structure. (C) Sewn ripstop nylon body with integrated magnetic tip. (D) Coated nylon robot during underwater eversion and retraction. E) Large‐scale coated‐nylon robot and industrial‐pipe deployment. (A) (ii) Reproduced under the terms of the Creative Commons Attribution‐Non Commercial‐No Derivatives 4.0 International License [64]. Copyright 2025, The Authors. (B) Reproduced with permission [65]. Copyright 2020, IEEE. (C) Reproduced with permission [66]. Copyright 2025, Mary Ann Liebert, Inc. (D) Reproduced with permission [67]. Copyright 2019, IEEE. E) Reproduced with permission [68]. Copyright 2025, IEEE.

In fluid‐driven vine robots, nylon is commonly used as a woven fabric rather than as a homogeneous film. Ripstop fabrics contain reinforcing yarns in the warp and weft directions. These yarns carry pressure‐induced membrane loads, restrict radial ballooning, and limit the propagation of local tears. The weave also introduces strong mechanical anisotropy. Loading along the yarn directions mainly stretches the fibers, whereas bias loading is accommodated through yarn rotation, crimp straightening, and in‐plane shear. Uncoated woven nylon is not sufficiently gas‐retentive for a pressure‐driven body because gas can pass through the textile pores, and stitched seams add leakage paths. Nylon is therefore commonly combined with TPU, polyurethane, silicone, or hybrid coatings. The yarn network provides reinforcement and tear resistance, while the coating forms the pressure barrier, modifies friction, protects the fibers, and supplies a joining interface. For example, a TPU‐coated ripstop nylon exhibited effective moduli of approximately 215 and 201 MPa along its principal directions. A silicone‐coated nylon showed moduli of 96.5 and 120 MPa in these directions but only 4.05 MPa at 45° [71]. Yarn alignment can therefore stabilize the inflated tube, whereas bias‐oriented regions can remain compliant for folding, bending, or torsion. The relevant material system is consequently a laminate rather than nylon alone.

These benefits introduce a trade‐off at the eversion front. Higher yarn density and thicker fabrics improve burst and tear resistance, but they also increase bending resistance, stored‐body volume, and the pressure required to reverse the wall through a small tip radius. Favorable tribological behavior has been reported for nylon because sliding can generate a transfer film that reduces subsequent friction [72]. However, this mechanism should not be generalized to all nylon‐based robots. In coated textiles, the sliding surface is usually the coating rather than the polyamide yarn. Eversion and retraction therefore depend on the coating chemistry, surface roughness, and other factors.

TPU‐coated nylon can be heat‐sealed or ultrasonically welded because the thermoplastic coating softens and fuses. This facilitates the fabrication of long tubes, steering chambers, preformed folds, and branched structures. Silicone‐coated nylon generally requires a compatible adhesive because silicone cannot be joined through conventional thermoplastic remelting. Naclerio and Hawkes [65] used silicone‐ and urethane‐impregnated ripstop nylon bonded with a silicone adhesive (Figure 5B). The resulting single‐layer fabric pneumatic muscle remained foldable when depressurized, exhibited less than 1% hysteresis, and exceeded 100 000 cycles. Although this was not a complete vine‐robot lifetime test, it showed that matching the coating and adhesive while eliminating bladder–sleeve sliding can reduce interfacial friction and wear.

Manufacturing quality can be as important as the nominal textile. Weld temperature, dwell time, pressure, overlap width, coating orientation, and surface contamination affect seam strength and leakage [73]. A narrow or overly stiff weld may resist pressure but concentrate stress near the heat‐affected boundary. Adhesive joints may remain compliant and repairable but can peel under repeated folding. Long‐term reliability is further limited by coating cracking, abrasion, delamination, pinholes, yarn damage, and frictional jamming. Polyamides also absorb moisture, primarily through less ordered regions. Absorbed water modifies the hydrogen‐bonding network, increases chain mobility, and commonly reduces stiffness and dimensional stability [74, 75]. Repeated loading may also reorganize the fiber microstructure and alter modulus or hysteresis before visible failure [76]. Material screening should therefore include cyclic eversion, inversion, pressure‐decay, abrasion, and wet–dry conditioning rather than monotonic strip tests alone.

The development of nylon‐based growing systems illustrates the transition from woven hoses to coated functional laminates. Kiryu et al. [36] developed the plant‐inspired Grow‐hose‐I using a nylon‐containing hose, providing an early example of a pneumatically extending device. Kim et al. [66] later fabricated a vine‐robot body from sewn ripstop nylon, sealed the seam with silicone adhesive, and integrated a magnetic tip for externally applied steering forces and torques (Figure 5C). More broadly, recent magnetic soft‐material platforms demonstrate shape programming and in situ magnetization reprogramming [77, 78]. These latter studies are not nylon‐based vine‐robot demonstrations, but they illustrate functional‐material strategies that could be transferred to compliant growing systems.

Coated fabrics extended this strategy to more demanding environments. Coad et al. [11] selected 0.15 mm TPU‐coated ripstop nylon for a 7.5 m field robot operating at pressures up to 21 kPa because rocky deployment required greater damage tolerance than LDPE could provide. Haggerty et al. [79] used a silicone–urethane‐treated ripstop nylon body with an internal steering–reeling mechanism. The measured inversion threshold was 0.30 ± 0.05 N, whereas sliding between the mechanism and fabric required 0.65 ± 0.05 N. This comparison shows that interface friction can dominate retraction even when the textile is highly flexible. Luong et al. [67] similarly found that a heat‐sealable nylon taffeta generated excessive friction and instead selected silicone‐ and polyurethane‐coated ripstop nylons for hydraulic underwater growth and retraction (Figure 5D). Thus, neither “nylon” nor “coated nylon” is sufficient as a material specification; coating and interface behavior must also be reported.

Coated nylon also enables extreme geometric scaling. Heap et al. [68] fabricated a 33 m long, approximately 1 m diameter robot from 0.08 mm silicone‐coated ripstop nylon with an areal density of 50.8 g m^−2^. The robot navigated a 24 m industrial pipe (Figure 5E). A continuous sliding interface prevented loose stored fabric from jamming at the tip mechanism. This system shows that lightweight textiles can support very large deployable bodies, but also that material handling and friction become increasingly important as the stored fabric volume grows.

Overall, nylon‐based vine‐robot bodies should be understood as hierarchical composite structures. Polyamide chemistry governs fiber strength, moisture sensitivity, and cyclic behavior. Drawing and weaving provide directional reinforcement and tear control. The coating supplies pressure retention, environmental protection, frictional characteristics, and joining capability, while the seam converts the laminate into a pressure vessel. This hierarchy offers greater durability than unsupported thin films but introduces additional bending resistance, process variability, moisture sensitivity, and interfacial failure modes. Future studies should report the polyamide grade, yarn density and orientation, coating chemistry and thickness, seam process, friction conditions, pressure‐decay performance, and cycles to leakage or failure. Such information is necessary to connect material design with growth pressure, steering accuracy, retraction stability, and field service life.

### 2.2 Material Requirements for Phase‐Transition and Deposition‐Enabled Growth

#### 2.2.1 Low‐Melting‐Point Alloys (LMPAs)

Low‐melting‐point alloys (LMPAs) are fusible metallic alloys that undergo solid–liquid transitions at temperatures far below those of conventional structural metals [83, 84]. For vine robotic systems, relevant compositions typically transition between approximately 30°C and 100°C. LMPAs are usually encapsulated within compliant structures and repeatedly switched between solid and liquid states. Local melting creates a deformable region for extension or steering, whereas resolidification restores load‐bearing capability. Their main value in vine robots is therefore the spatial and temporal programming of mechanical state. Alloy composition determines the balance among transition temperature, mechanical performance, processability, and safety. Gallium‐based alloys can remain liquid near room temperature, but supercooling and surface oxides can complicate reliable solid‐state load‐bearing [84]. Bismuth‐, indium‐, and tin‐based fusible alloys are more suitable when the robot must remain rigid at room temperature. Representative examples include Rose's metal at approximately 98°C, Cerrolow‐117 at approximately 47°C, and lead‐ and cadmium‐free Field's metal at approximately 62°C [10, 81, 82]. Some formulations contain lead or cadmium. Biomedical designs must therefore consider both compositional toxicity and tissue‐facing temperature, while encapsulation must prevent leakage, provide electrical insulation, and tolerate repeated thermal cycling. These systems are therefore better regarded as phase‐transition‐enabled or hybrid growth strategies rather than material deposition.

For vine robots, the distinctive contribution of LMPAs is their ability to create a movable compliant zone within an otherwise load‐bearing body [10, 81, 82]. A distal segment can be softened for extension and steering, while the solid proximal region preserves the previously formed trajectory and transmits loads. After the distal region is resolidified, the phase‐transition zone can be advanced. This sequential process produces a functional soft‐tip/stiff‐base organization, in which compliant regions support exploration and rigid regions provide structural support. Local phase control can therefore decouple navigation from load bearing and enable follow‐the‐leader motion, shape locking, force transmission, and buckling suppression. These functions arise from the change in load‐bearing mechanism across the solid–liquid transition. In the solid state, the continuous metallic core carries bending and shear loads. After melting, the mechanical response is governed mainly by the encapsulating elastomer, embedded heater, internal pressure, and any remaining solid fraction. The effective stiffness ratio is therefore a system‐level property rather than an intrinsic alloy constant. It depends on the core diameter, shell thickness, cross‐sectional geometry, filling continuity, and completeness of melting. Partial transition can produce intermediate stiffness and reduce the repeatability of shape control. Earlier LMPA‐filled fibers and continuum devices established the feasibility of reversible stiffness regulation in slender soft structures (Figure 6A) [80, 85]. Achieving this behavior reliably requires control of both phase‐transition thermodynamics and heat transfer. A lower transition temperature reduces the required thermal excursion, but does not necessarily provide faster or more energy‐efficient switching. Heating and cooling also depend on alloy volume, latent heat, thermal conductivity, heater–alloy contact, encapsulation thickness, and heat exchange with the environment [82, 83]. Excessive heating can cause thermal spillover and unintentionally soften neighboring rigid regions, whereas cooling may limit the growth‐cycle rate because stiffness is recovered only after sufficient heat removal.

**FIGURE 6:**
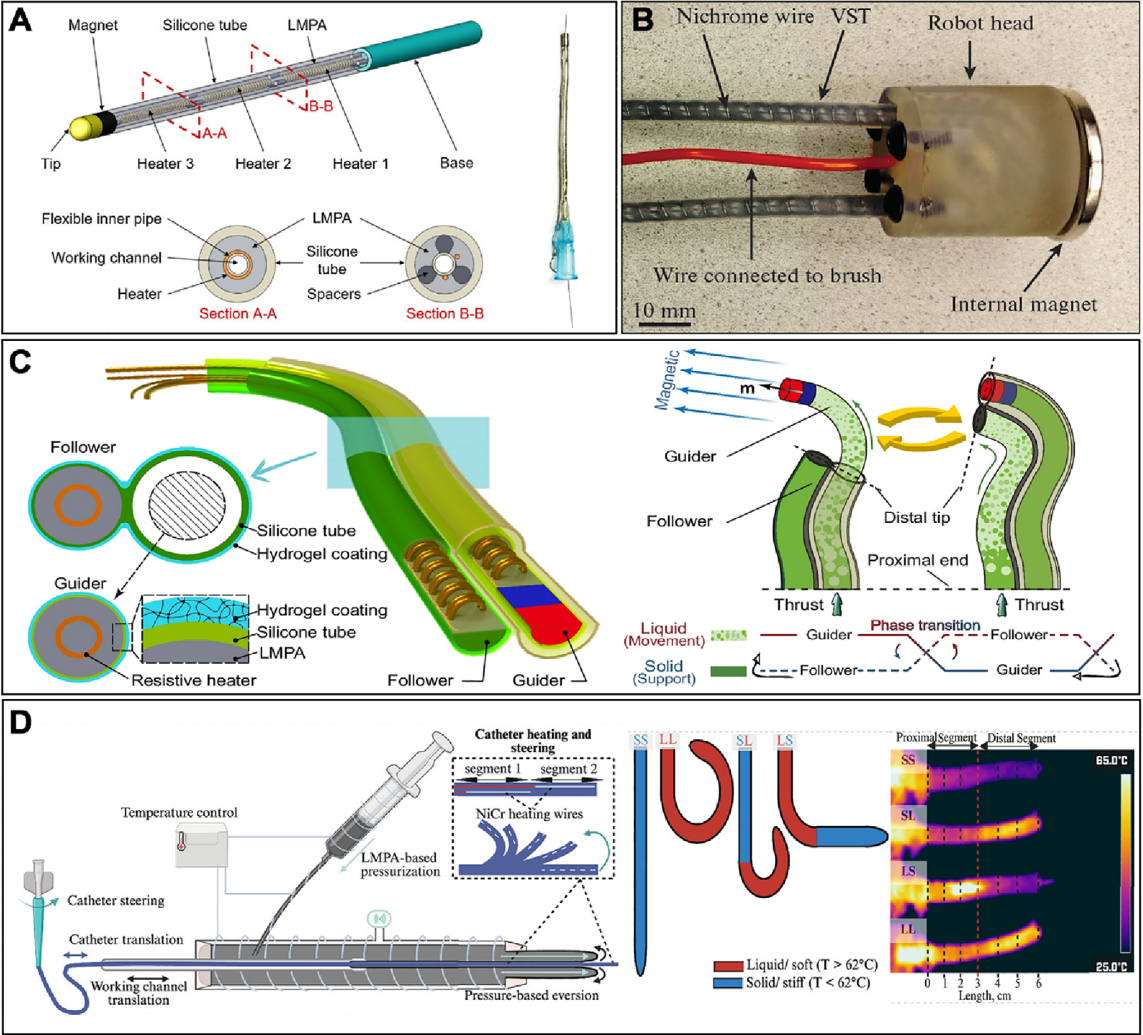
LMPA‐enabled variable stiffness and phase‐transition growth. (A) Magnetically guided LMPA continuum device with segmented heaters and a working channel. (B) Rose's metal‐filled variable‐stiffness tube with distal magnetic steering. (C) Cerrolow‐117‐based Guider–Follower architecture for follow‐the‐leader growth. (D) Field's metal‐driven everting robot with spatially programmed liquid and solid segments. (A) Reproduced with permission [80]. Copyright 2019, Wiley‐VCH GmbH. (B) Reproduced with permission [10]. Copyright 2024, IEEE. (C) Reproduced under the terms of the Creative Commons Attribution 4.0 International License [81]. Copyright 2024, The Authors. (D) Adapted under the terms of the Creative Commons Attribution 4.0 International License [82]. Copyright 2025, The Authors, published by Wiley‐VCH GmbH. LMPA, low‐melting‐point alloy.

Venkiteswaran et al. [10] demonstrated this principle using a Rose's‐metal‐filled variable‐stiffness tube (Figure 6B). The alloy was encapsulated in a 3 mm‐inner‐diameter silicone tube and heated by a 0.12 mm nichrome wire. Selective melting reduced distal bending resistance and enabled magnetic guidance, while the solid proximal region supported the deployed body. Repeated heating, extension, and cooling advanced the compliant zone. The system showed an effective stiffness ratio of approximately 83, with reported stiffness values of 1.304 N m^−1^ in the solid state and 0.016 N m^−1^ in the liquid state. However, the high transition temperature and lead‐containing composition of Rose's metal constrain thermal safety and biomedical translation. Mao et al. [81] used Cerrolow‐117 to realize follow‐the‐leader growth through two coordinated phase‐transition components, termed the Guider and the Follower (Figure 6C). Each contained an LMPA core and resistive heater within a silicone tube, while a coating reduced sliding friction. The Guider was softened, advanced, and magnetically steered as the solid Follower retained the deployed path. After the Guider resolidified, the Follower was melted and advanced along the same trajectory, and the two components exchanged roles. This alternating sequence separated guidance from support and enabled three‐dimensional path retention without whole‐body sliding. Excessive heating, however, could soften both components and destroy the locked configuration, making independent temperature regulation essential. Al Harthy et al. [82] further showed that an LMPA can serve as both the actuation medium and the variable‐stiffness element of a vine‐everting robot. Field's metal pressurized and everted the body in its liquid state, then solidified to reinforce selected segments. A steerable catheter provided localized heating, allowing compliant and rigid regions to be programmed along the body. The robot achieved an approximately 43‐fold stiffness ratio and a 15‐fold increase in force output (Figure 6D). This architecture consolidates fluidic actuation and structural reinforcement within the same phase‐change material. The robot can therefore remain compliant during navigation and locally stiffen for force delivery, payload support, or buckling resistance. Precise thermal localization remains essential to avoid softening neighboring rigid regions.

Long‐term performance depends on the complete alloy–heater–encapsulation system. Repeated phase transitions may promote microstructural changes, void formation, leakage, or local fracture, while heaters and electrical leads can create stiffness discontinuities and stress concentrations. High alloy density also increases self‐weight. During miniaturization, the shell and heater occupy a larger fraction of the cross‐section, reducing the alloy core's contribution to stiffness change. Miniaturized designs must balance alloy volume against the space required for insulation, connections, and working channels. Reliability should be assessed through repeated thermal–mechanical cycling, leakage testing, and stiffness‐drift measurements. Remote heating may eventually reduce dependence on wired components. Photothermally responsive liquid‐metal/polymer composites have demonstrated near‐infrared‐triggered deformation and stiffness‐related functions in other soft robotic systems [86]. Optical or inductive approaches could provide more localized phase control, but these capabilities have not yet been robustly demonstrated in vine robots.

Overall, LMPAs provide a distinctive material strategy for coupling growth, local compliance, and structural maturation. Their key advantage is the ability to move a soft region through an otherwise load‐bearing body and retain stiffness after solidification without continuous power input. This behavior is valuable for guided extension, follow‐the‐leader navigation, shape retention, buckling suppression, and force delivery in confined environments. The main trade‐offs are thermal response time, density, encapsulation complexity, possible toxic constituents, and unintended heating of adjacent regions. Future designs should co‐optimize alloy composition, transition temperature, thermal properties, geometry, heater architecture, encapsulation, and environmental heat transfer. These coupled factors, rather than melting point alone, determine whether LMPA‐enabled growth can achieve reliable, safe, and scalable performance.

#### 2.2.2 Thermoplastic Deposition Materials: PLA and PCL

In contrast to low‐melting‐point alloys, thermoplastic polymers enable growth through localized melting, deposition, and solidification rather than reversible phase switching. A filament is transported from the base to the growing head, heated into a flowable state, and deposited along a prescribed path. The deposited material then cools to form the load‐bearing robot body [89]. Material selection therefore determines not only post‐growth stiffness, but also growth rate, energy demand, dimensional accuracy, and printing reliability. An early precursor to thermoplastic deposition‐based growth was reported by Sadeghi et al. [35]. Their root‐inspired device fed a polypropylene filament from an external spool to a rotating tip, where it formed a hollow tube and generated penetration force while the mature body remained stationary. Although it lacked the localized melting and fusion later used for PLA printing, it established three key principles: base‐to‐tip material transport, apical construction, and rapid structural maturation behind the growing zone. Because quantitatively comparable tip‐growing studies remain limited for many other printable feedstocks, the discussion below focuses on PLA and PCL, for which the available literature provides clear links among processing, structure, and robot‐level performance.

Poly(lactic acid) (PLA) is an aliphatic polyester produced from lactic acid, which can be obtained from renewable carbohydrate feedstocks [90]. Its properties depend on molecular weight, stereoregularity, crystallinity, additives, and thermal history. Representative grades exhibit a glass‐transition temperature of approximately 55°C–65°C, a melting range of about 150°C–180°C, a Young's modulus of roughly 3–4 GPa, and a tensile strength of 40–60 MPa [90, 91]. Tip‐deposited structures contain interfaces between layers, and their properties also depend on layer thickness, porosity, printing direction, and cooling history.

The high stiffness of solid PLA is advantageous after deposition. It enables thin printed tubes to support the growing head, preserve the programmed geometry, and carry external loads. However, PLA is comparatively brittle and has limited strain to failure. Weakly bonded layers can therefore crack during bending, obstacle contact, or long‐term loading. The robot‐scale response is governed by both the polymer and the printed architecture.

Thermal processing directly controls growth. Increasing the extrusion temperature lowers the melt viscosity of PLA and generally improves material flow, raster coalescence, and interlayer bonding [92, 93]. However, the deposited filament must remain only partially molten. If it is too fluid, it cannot resist external pressure or support the growing head; if it is too solid, adjacent layers cannot fuse reliably [34]. Excessive heating also prolongs cooling and increases the thermal load on the motors and electronics. Conversely, insufficient heating causes poor flow, void formation, and weak interlayer interfaces [87]. Feed speed, plotting speed, nozzle temperature, and cooling rate should therefore be optimized as a coupled process window rather than as independent parameters.

Building on early deposition architecture, Sadeghi et al. [34] subsequently demonstrated tip‐based PLA deposition in a root‐inspired robot. The system constructed a tubular body in situ and generated steering through differential material deposition. Local differences in deposited volume produced curvature, allowing the feedstock to serve simultaneously as the structural body and the steering medium. A subsequent study exploited the transient compliance of recently deposited PLA for passive obstacle avoidance [94]. Before complete cooling, the soft growth zone deformed upon contact with an obstacle. It then solidified and retained the contact‐defined path. The same thermal transition thus provided temporary compliance at the tip and permanent support behind it.

Del Dottore et al. further developed this strategy in FiloBot, which integrates a sensorized additive‐manufacturing head with environmental perception and closed‐loop growth control (Figure 7A) [9, 95]. PLA filament is supplied from the base, melted at the tip, and deposited into a tubular stem. The robot adjusts feeding, plotting, temperature, and cooling according to gravity, light, and structural requirements. By varying layer thickness, material distribution, and processing conditions, the system can produce lightweight structures for rapid growth or stiffer structures for self‐support and gap crossing. FiloBot demonstrates how a fixed thermoplastic feedstock can generate different robot‐scale properties through programmed deposition.

**FIGURE 7:**
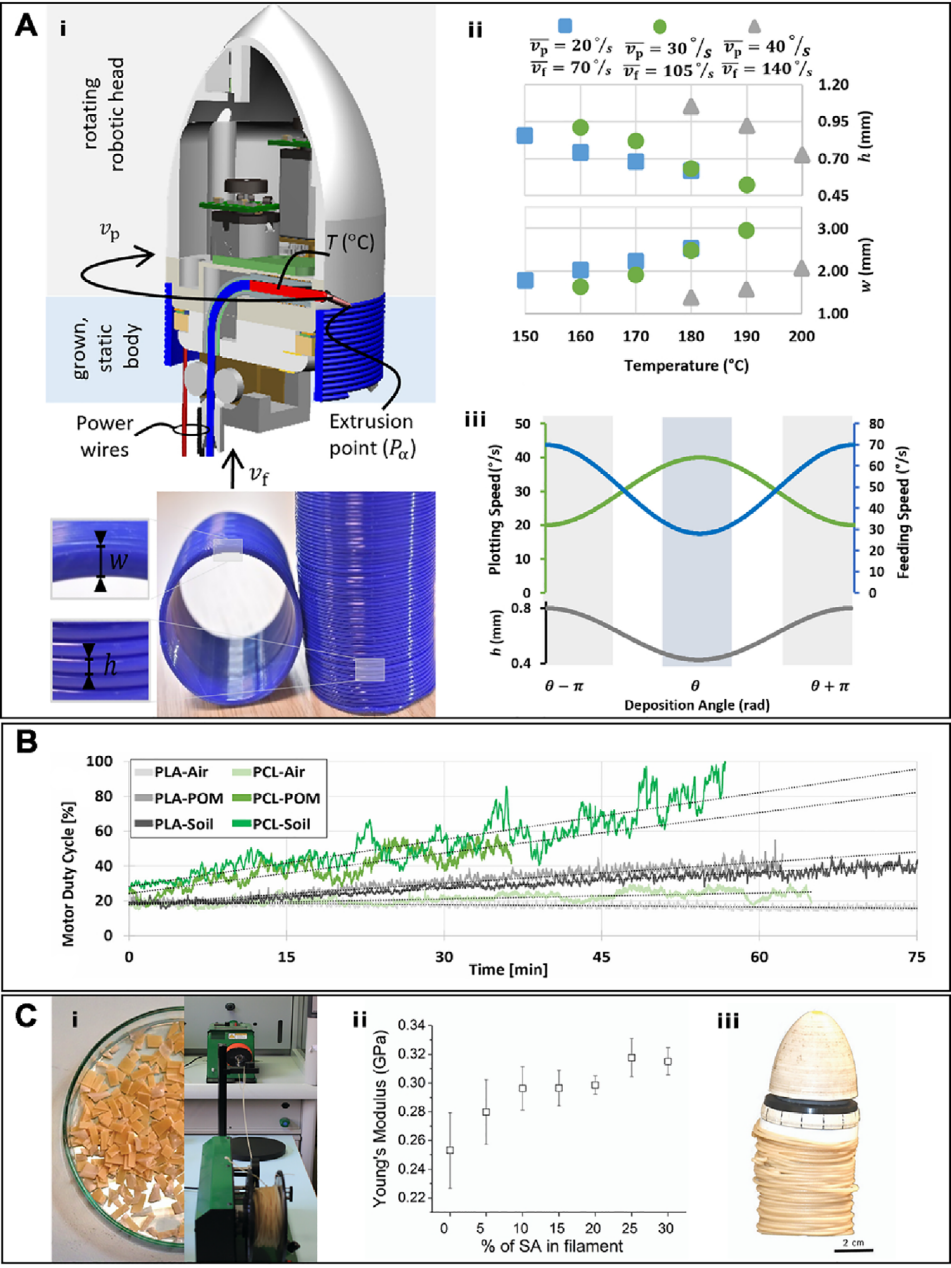
Thermoplastic deposition strategies for growing robots. (A) (i) FiloBot growth head and body structures, (ii) deposition parameters, (iii) programmed variation of layer geometry during PLA growth. (B) Comparison of extrusion‐motor load during PLA and PCL deposition in air, polyoxymethylene granules, and soil. (C) (i) PCL/sodium alginate composite filament, (ii) composition‐dependent Young's modulus, (iii) deposited root‐like body. (A) Adapted with permission [9]. Copyright 2024, AAAS. (B) Reproduced under the terms of the Creative Commons Attribution 4.0 International License [87]. Copyright 2023, The Authors. (C) Reproduced under the terms of the Creative Commons Attribution 3.0 Unported License [88]. Copyright 2020, The Authors. PCL, polycaprolactone; PLA, polylactic acid; POM, polyoxymethylene; SA, sodium alginate.

PLA nevertheless imposes clear constraints. Its relatively high processing temperature increases heater power and thermal‐management requirements. Growth is limited by melting, deposition, and cooling, making it slower than pressure‐driven eversion. The solidified body is also not inherently retractable. Removal generally requires cutting, remelting, dissolution, or degradation. This limits the applications of the PLA vine robot. PLA is therefore suitable when permanent shape retention and post‐growth load bearing are more important than rapid or reversible deployment.

Polycaprolactone (PCL) provides a complementary thermoplastic option. It is a semicrystalline aliphatic polyester with a glass‐transition temperature near −60°C and a melting range of approximately 56°C–65°C [96]. Compared with PLA, PCL generally exhibits a lower modulus and greater extensibility. It is therefore softer, more ductile, and less susceptible to brittle fracture at room temperature [90]. Its lower melting temperature reduces the temperature required at the growing head and can decrease heater demand and thermal exposure of nearby components. These advantages are offset by slower solidification and lower solid‐state stiffness. Because molten PCL is only moderately hotter than the surrounding environment, the deposited material can remain soft and tacky for a relatively long period [87]. This may increase friction against the plotting mechanism, cause filament slippage, and produce irregular layers. The newly formed body also provides less immediate support for the growing head. A lower melting point therefore does not necessarily result in faster or more reliable growth.

A direct comparison of PLA and PCL in a root‐inspired burrowing robot illustrates this trade‐off [87]. PCL reduced the processing temperature from approximately 180°C to 60°C, but produced greater internal friction and less stable motor loading. In soil, incomplete cooling caused sticking, slippage, and irregular deposition, whereas PLA formed a more stable structure under the tested conditions (Figure 7B). Thermoplastic selection cannot therefore be based on melting temperature alone. Melt viscosity, tackiness, cooling rate, solid‐state stiffness, and friction with the printing mechanism must also be considered.

The low processing temperature and chemical versatility of PCL may nevertheless facilitate functional feedstocks. Liakos et al. blended PCL with sodium alginate to produce extrudable composite filaments (Figure 7C) [88]. The alginate phase increased stiffness and introduced heavy‐metal adsorption capability. A plant‐inspired growing robot deposited the composite into a tubular structure while retaining its adsorption function. This example extends material‐deposition growth beyond structural construction. The printed body can also act as an environmental interface for adsorption, sensing, or remediation.

Overall, PLA and PCL occupy different regions of the thermoplastic design space. PLA provides high post‐deposition stiffness, rapid shape retention, and favorable load‐bearing capability, but requires higher processing temperatures and is more susceptible to brittle interlayer failure. PCL offers low‐temperature processing, ductility, and compatibility with functional additives, but its lower stiffness, tackiness, and slow cooling can reduce deposition stability. Their suitability depends on the required balance among thermal exposure, growth speed, structural permanence, environmental interaction, and printing reliability. For thermoplastic growing robots, the design variable is not the polymer alone, but the coupled system of feedstock chemistry, melt flow, deposition parameters, cooling conditions, and printed architecture.

### 2.3 Comparative Discussion of Material Strategies

The asymmetry between fluid‐driven eversion and in situ body formation should not be interpreted as a difference in material importance. Eversion has been demonstrated across a broader range of robot scales and applications. Its prefabricated bodies can also be compared using relatively common quantities such as wall thickness, diameter, pressure, growth length, and growth speed. Deposition‐based systems are represented by fewer platforms, and their performance depends strongly on the complete processing route. The relevant material description therefore extends from feedstock chemistry to melt rheology, deposition geometry, interfacial bonding, cooling history, and the architecture created during growth. Phase‐transition systems based on LMPAs occupy an intermediate position because they alter mechanical state locally without constructing the body through conventional additive deposition.

Table 1 compiles representative vine‐inspired growing robots reported in the literature, including their growth mode, body material, robot diameter, maximum reported growth length, growth pressure, growth speed, publication year, and reference. The table provides a common system‐level basis for comparing fluid‐driven eversion and material‐deposition growth across different material and architectural strategies. Because these parameters were reported under different experimental conditions and are not available for every system, the compiled values should be interpreted as representative reported performance rather than as standardized benchmarks. The quantitative data in Table 1 are further reorganized in Figure 8 to visualize the relationships between robot diameter and growth length, growth speed, and growth pressure. Accordingly, Figure 8 summarizes the reported system‐level performance across the literature rather than the intrinsic capability of individual materials. Eversion systems cover a broad range of diameters and growth lengths (Figure 8A) and generally exhibit higher reported growth speeds than deposition‐based systems (Figure 8B), consistent with the compact storage and low moving mass of thin‐film and coated‐textile bodies. These differences, however, also depend on pressure and flow supply, body geometry, material‐storage architecture, internal friction, and fabrication quality. Because several studies identify the body material only as PE, these data are grouped with those explicitly reporting LDPE as PE/LDPE, rather than treating them as separate material classes. Material categories represented by only a few comparable studies should therefore be regarded as indicative rather than as defining a material‐specific performance envelope. Figure 8C includes only systems in which fluidic driving pressure is an appropriate actuation parameter and should not be used for direct comparison across different growth mechanisms.

**TABLE 1:** Representative structural dimensions and growth performance of vine‐inspired robots.

Growth mode	Robot body material(s)	Robot diameter (mm)	Maximum growth length (m)	Growth pressure (kPa)	Growth speed (mm s^−1^)	Year	Refs.
Fluid‐driven eversion	Polyethylene film	1.8–360	72	20^a^	10 000 (max.)	2017	[8]
Fluid‐driven eversion	LDPE film	4	—	15.5–31	—	2017	[12]
Fluid‐driven eversion	Ripstop nylon outer layer + LDPE inner tube	27	—	—	—	2018	[13]
Fluid‐driven eversion	Thin‐walled polyethylene tube	32	—	14	—	2018	[14]
Fluid‐driven eversion	LDPE	48	—	13.8	—	2019	[23]
Fluid‐driven eversion	Silicone‐coated ripstop nylon	48	0.80	10	10	2019	[67]
Fluid‐driven eversion	LDPE	50	10	14	100	2020	[11]
Fluid‐driven eversion	TPU‐coated ripstop nylon	75	7.5	21	100	2020	[11]
Fluid‐driven eversion	Silicone‐coated ripstop nylon	23	—	34.5	—	2020	[128]
Fluid‐driven eversion	Silicone–polyurethane‐impregnated ripstop nylon (71 µm)	75	2.4	—	—	2021	[79]
Fluid‐driven eversion	Silnylon (silicone/polyurethane‐impregnated ripstop nylon)	100	—	—	—	2021	[101]
Fluid‐driven eversion	Thin‐walled polyethylene	76.4	1.15	10^b^	—	2021	[107]
Fluid‐driven eversion	40D polyurethane‐coated ripstop nylon	3.5–5	—	200–300	—	2021	[134]
Fluid‐driven eversion	LDPE	66	1.15	—	—	2021	[148]
Fluid‐driven eversion	PE	79.8	0.85	—^c^	—	2023	[104]
Fluid‐driven eversion	TPU‐coated 70D ripstop nylon	80	2	3.7	—	2023	[108]
Fluid‐driven eversion	LDPE; TPU‐coated ripstop nylon	33	—	11.5–23.7	37.5–100	2023	[113]
Fluid‐driven eversion	40D TPU‐coated ripstop nylon	108	2.3	7	—	2023	[114]
Fluid‐driven eversion	LDPE substrate + magnetic‐particle/silicone coating	8	0.25	12	—	2024	[98]
Fluid‐driven eversion	Ripstop nylon fabric	63.6	—	1.2	70	2024	[115]
Fluid‐driven eversion	TPU‐coated ripstop nylon	127^d^	7.6	—	—	2024	[145]
Fluid‐driven eversion	TPU‐coated ripstop nylon	133^d^	—	—	41.7	2024	[145]
Fluid‐driven eversion	PE film (0.05 mm)	58	—	4.2–10	15	2025	[26]
Fluid‐driven eversion	Ripstop nylon fabric	25	—	30	—	2025	[66]
Fluid‐driven eversion	Silicone‐coated nylon	1000	33	—	≈25^e^	2025	[68]
Fluid‐driven eversion	TPE film (Stretchlon 200, 38 µm)	5	0.145	35	—	2025	[109]
Fluid‐driven eversion	LDPE sheath + Field's‐metal LMPA	6.4	0.060	10^f^	—	2025	[82]
Phase‐transition‐assisted tip growth	Cerrolow‐117 LMPA core + silicone encapsulation (hydrogel‐coated)	≈3–4	0.40^g^	—	—	2024	[81]
Material‐deposition growth	PLA	50	—	—	0.0667	2017	[34]
Material‐deposition growth	PLA	46	—	—	0.0527–0.0967	2018	[95]
Material‐deposition growth	PLA	48	0.27	—	0.05–0.0667	2020	[94]
Material‐deposition growth	PLA + PCL	40	—	—	—	2023	[87]
Material‐deposition growth	PLA	40	0.50	—	0.0333–0.1167	2024	[9]

^a^ For Ref. [8], the reported diameter range, maximum length, maximum speed, and the 20 kPa pressure point correspond to different robot configurations; 20 kPa was reported for *D* = 48 mm.

^b^ For Ref. [107], 10 kPa is a fluid‐chamber simulation condition rather than an experimentally reported growth pressure.

^c^ For Ref. [104], the reported 140 kPa value is a maximum pressure tolerance/safety limit rather than a growth pressure and is therefore shown as “‐” in the growth‐pressure column.

^d^ The two Ref. [145] rows represent different SPROUT configurations; the 127 mm configuration provides the 7.6 m length, whereas the 133 mm configuration provides the 41.7 mm s^−^^1^ speed.

^e^ For Ref. [68], ≈25 mm s^−^^1^ is an estimate from the published figure rather than a directly tabulated speed.

^f^ For Ref. [82], 10 kPa is the reported relative pressure at which eversion initiates; pressures of 35 kPa or higher led to bursting.

^g^ For Ref. [81], ≈3–4 mm is the reported maximum cross‐sectional size. The 0.40 m value corresponds to the ≈400 mm robot used for the experimental demonstrations; the paper notes that the architecture can potentially extend to meter‐scale lengths.

Abbreviations: LDPE, low‐density polyethylene; LMPA, low‐melting‐point alloy; PCL, polycaprolactone; PE, polyethylene; PLA, Poly(lactic acid); TPE, thermoplastic elastomer; TPU, thermoplastic polyurethane. A hyphen (‐) indicates that the corresponding value was not reported or was not retained as the requested metric.

**FIGURE 8:**
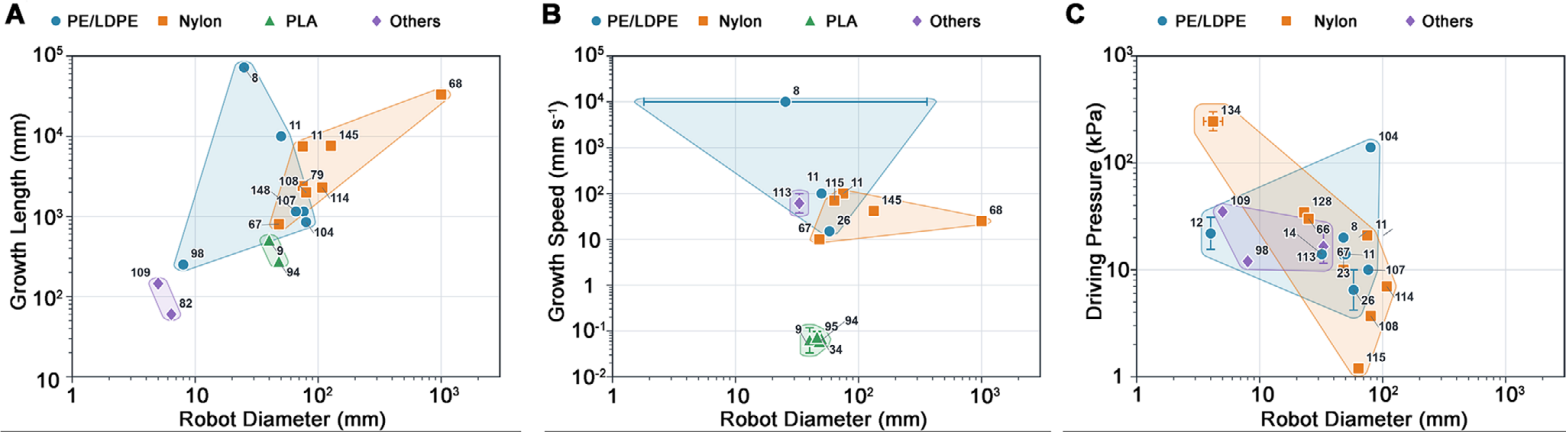
Literature‐derived system‐level performance landscape of representative vine robots. (A) Growth length as a function of robot diameter. (B) Growth speed as a function of robot diameter. (C) Fluidic driving pressure as a function of robot diameter. For entries reported as ranges, the marker is placed at the geometric midpoint and the full reported interval is shown by a whisker. Shaded envelopes are descriptive visual guides constructed separately for each material category by taking the convex hull in log10(diameter)–log10(response) space, expanding it outward by 0.075 log unit, and applying three Chaikin corner‐cutting iterations. They should not be interpreted as confidence regions, probability densities, prediction intervals, or intrinsic material‐property domains. The PE/LDPE category combines studies reporting LDPE with studies specifying only PE. Panels A and B include PE/LDPE, Nylon, PLA, and Others and encompass multiple growth strategies; panel C includes PE/LDPE, Nylon, and Others and retains source‐table entries with a positive numeric fluidic‐pressure value. Both axes are logarithmic. Others denotes systems assigned to the remaining category in the source table. LDPE, low‐density polyethylene; LMPA, low‐melting‐point alloy; PE, polyethylene; PLA, polylactic acid.

Material‐deposition growth introduces additional constraints that are not captured by pressure‐based comparisons. Thermoplastic extension requires filament transport, melting, extrusion, interlayer fusion, and sufficiently rapid solidification at the tip. Higher temperature can improve flow and bonding but may delay shape stabilization. Lower‐melting materials reduce thermal demand but can remain tacky for longer, increasing friction or deposition irregularity, as observed for PCL relative to PLA. Growth rate is thus governed by a coupled thermal‐rheological process window. Feedstock buckling, nozzle force, tip mass, dimensional accuracy, and environmental heat transfer become increasingly important during miniaturization or growth in contact‐rich media. The deposited body is also usually structurally permanent and lacks the inherent inversion‐based retraction available to eversion robots, which complicates retrieval and reuse.

LMPA‐enabled systems present a different trade‐off. Local melting can create a compliant growth or steering zone, while resolidification restores load‐bearing capacity. This enables shape locking and variable stiffness, but requires thermal localization, encapsulation, leakage prevention, and stable thermal–mechanical cycling. Direct one‐to‐one comparison with thin‐film eversion or thermoplastic deposition is therefore incomplete.

From a bioinspiration perspective, eversion reproduces the functional outcome of tip extension. Deposition more closely resembles biological apical growth because new structural material is incorporated near the growing tip. Neither strategy is universally superior. Eversion favors rapid, long‐range, and potentially reversible deployment; deposition favors in situ shape creation and persistent structures; and phase‐transition systems provide local mechanical‐state programming. Material selection should therefore be based on task‐specific trade‐offs rather than a single performance metric.

## 3 Materials and Mechanisms for Functional Capabilities in Vine Robots

The growth mode of vine robots is intrinsically coupled to the structural and functional materials that form, deploy, and support the body. Tip growth, however, provides only the basic mode of extension and is not sufficient for efficient and flexible operation in complex, dynamic, and unstructured environments [11]. Natural vines complement apical growth with directional responses to light and contact, twining and attachment, branching, and spatially graded stiffening through tissue maturation. In robots, these behaviors arise from the coordinated design of material properties, structural architectures, interfaces, and localized mechanisms rather than from a single actuator class. Steering, variable stiffness, climbing and attachment, and branching have relatively direct biological analogies, whereas stable retraction and multifunctional tip integration primarily address engineering requirements for recoverability and task execution. Accordingly, this section organizes the literature by functional capability and broadly by the strength of the available experimental evidence: controlled steering, stable retraction, multifunctional tip design, variable stiffness, climbing and attachment, and branching (Figure 9A–G). Across these functions, the central materials question is how compliance, stiffness, friction, adhesion, magnetic response, or phase state can be redistributed without obstructing eversion or tip‐localized growth. These strategies have expanded the range of behaviors demonstrated by vine robots, although their robustness in uncontrolled environments remains unevenly evaluated.

**FIGURE 9:**
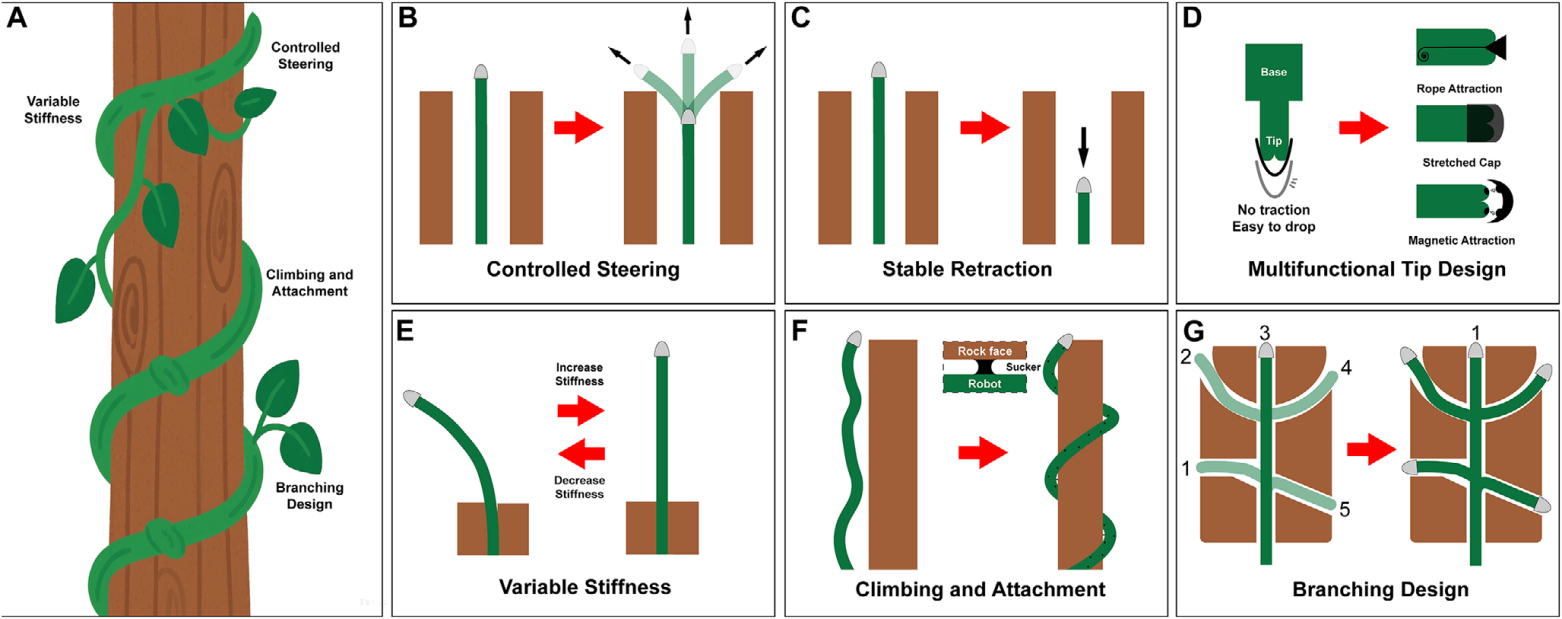
Material‐enabled functional capabilities in vine robots. (A) Biological analogy linking steering, variable stiffness, climbing and attachment, and branching. (B) Controlled steering. (C) Stable retraction. (D) Multifunctional tip design. (E) Variable stiffness. (F) Climbing and attachment. (G) Branching. Schematics were created by the authors.

### 3.1 Controlled Steering

Natural vines redirect growth in response to light and contact through phototropic and thigmotropic responses [4, 5]. Robotic analogues reproduce directional growth through differential bending and asymmetric deformation [102]. Steering in vine robots is not exclusively a materials problem; it can also be governed by mechanical architecture and control. Here, we focus on the material contribution: how local differences in strain, stiffness, friction, adhesion, or magnetic moment are created around the growing tip without obstructing eversion or tip‐localized deposition. Current strategies can be grouped into pressure‐driven systems shaped by films, fibers, seams, or interfaces; field‐ and stimulus‐responsive materials that generate force or contraction; and passive structures that encode curvature through preformed geometry or mechanical locking.

Pressure‐driven steering converts pneumatic input into differential contraction or extension. Greer et al. [103] placed three soft pneumatic artificial muscles near the tip. Their curvature depended on the compliance of the polyethylene tubes, radial constraint from rubber O‐rings, and pressure‐dependent chamber contraction. Deflation restored the tubes and enabled reversible bending. The later series pneumatic artificial muscles (sPAMs) design [23] retained this architecture while aligning extension with the local tip tangent, allowing steering without interrupting eversion. Coad et al. [11] used heat‐sealed pouch motors, in which film thickness, weld geometry, and seam stiffness governed inflation and axial shortening. These systems require low bending rigidity for eversion, but sufficient seam strength and dimensional stability for repeatable curvature.

Textile and interfacial materials can program anisotropy or selectively lock material release. Bianchi et al. [104] used Velcro strips to restrain one side of the body, converting symmetric pressure into asymmetric extension through frictional engagement. Jaén Mendoza et al. [97] developed a wrinkled composite containing an elastomeric TPU layer and inextensible Dyneema reinforcement (Figure 10A). The axial direction unfolded readily, whereas circumferential fibers suppressed radial expansion. This directional stiffness contrast enabled high‐curvature growth and lateral force generation while preserving pressure resistance.

**FIGURE 10:**
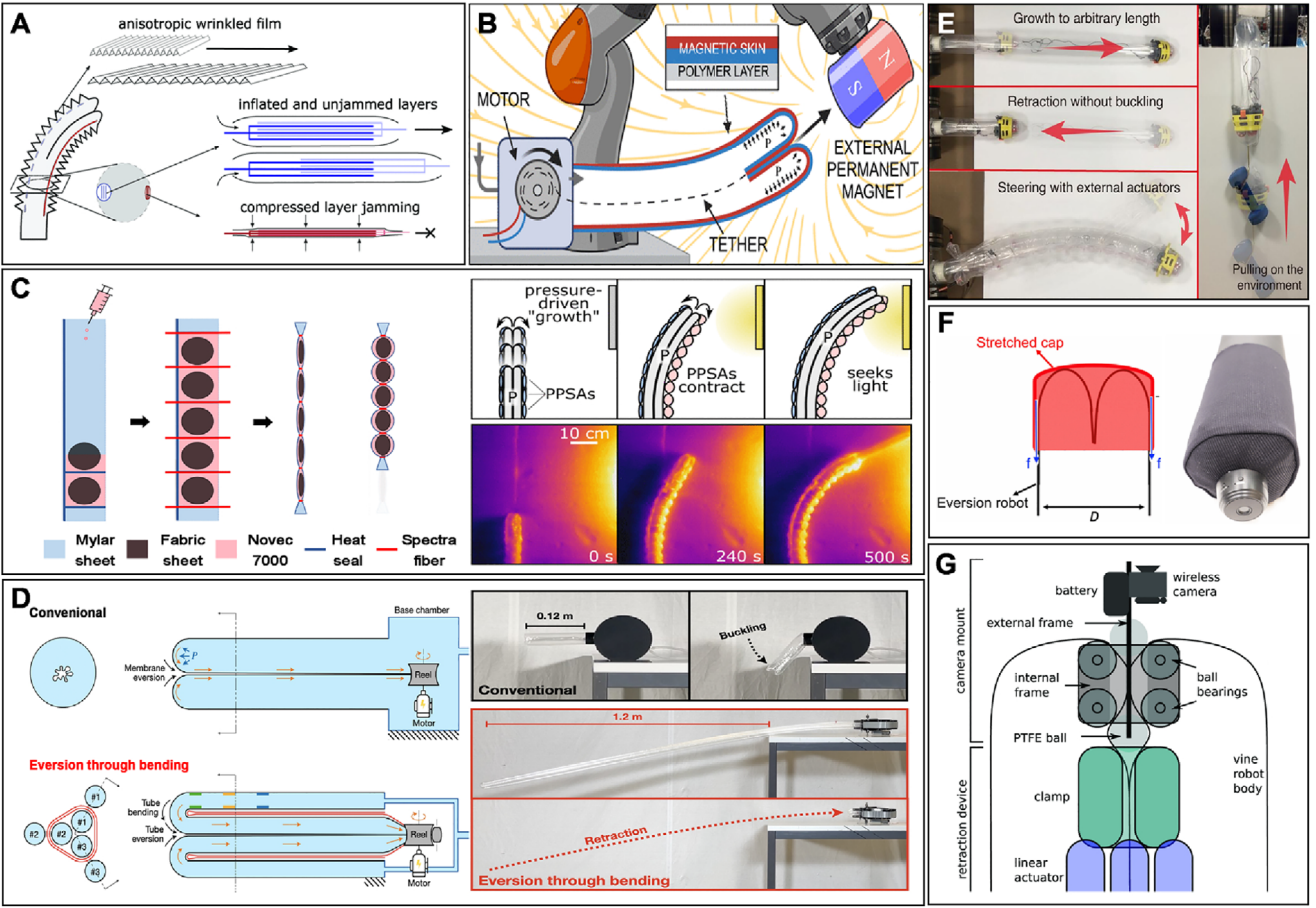
Representative material and structural strategies for steering, retraction, and multifunctional tip design. (A) Wrinkled anisotropic TPU/Dyneema composite for directional steering. (B) Orthogonally magnetized soft skin for distal magnetic steering. (C) Photothermal phase‐change pouch actuator for light‐controlled contraction. (D) Interconnected‐tube architecture for buckle‐free retraction. (E) Rolling‐interlock tip mount for growth, steering, and payload retrieval. (F) Soft textile cap for tip attachment. (G) Pneumatic retraction device with an internal camera mount. (A) Reproduced with permission [97]. Copyright 2024, IEEE. (B) Reproduced with permission [98]. Copyright 2024, IEEE. (C) Reproduced with permission [99]. Copyright 2024, IEEE. (D) Reproduced with permission [24]. Copyright 2024, IEEE. (E) Reproduced with permission [25]. Copyright 2020, IEEE. (F) Reproduced with permission [100]. Copyright 2023, IEEE. (G) Reproduced with permission [101]. Copyright 2021, IEEE. TPU, thermoplastic polyurethane.

Magnetic materials provide non‐contact steering, but their performance depends on particle distribution, magnetization, and matrix compliance [105, 106]. Magnetic latches locally retain or release stored material [107, 108]. More integrated designs can either localize magnetic functionality at the distal tip or distribute it through a soft magnetic skin. Kim et al. [66] integrated a permanent‐magnet tip capsule with a sewn ripstop‐nylon vine body, preserving a compliant body while enabling externally applied magnetic forces and torques. Davy et al. [98] reduced magnetic cancellation during eversion through orthogonal magnetization of NdFeB‐containing layers (Figure 10B). Increasing particle loading strengthens the magnetic response but also raises density and stiffness and may hinder eversion. Localizing the responsive phase at the tip reduces this conflict. Carbonyl‐iron suspensions produced strong field‐induced steering [109], whereas Liu et al. [57] embedded aligned NdFeB particles in an Ecoflex distal coating to steer a millimeter‐scale robot in a vascular phantom.

Stimuli‐responsive materials can combine environmental response with actuation. Qin et al. [26] attached shape‐memory‐alloy springs to one side of the body. Heating induced a martensite‐to‐austenite transformation and recoverable contraction, producing bending up to 80° [110]. Curvature and response time depend on transformation temperature, recoverable strain, actuation stress, heat transfer, and internal pressure. Deglurkar et al. [99] used a photothermal phase‐change actuator containing a light absorber and Novec 7000 (Figure 10C). Absorbed light vaporized the fluid, causing radial expansion and 20% axial contraction. Absorber efficiency, boiling point, latent heat, barrier properties, and pouch compliance jointly determined deformation. Both approaches reduce reliance on conventional steering hardware, but remain limited by energy consumption, cooling delay, and thermal compatibility.

Recent work further shifts steering from attached actuators toward a functionalized robot skin. Kim et al. [111] integrated thermally actuated liquid crystal elastomer (LCE) elements into thin robot skins for 3–7 mm‐diameter everting robots. Local contraction of the LCE generated multiple bends exceeding 100°, while the thin, compliant construction remained compatible with eversion. This approach strengthens the materials‐centered case for miniaturized steering because actuation is embedded in the skin itself, although the evidence remains limited to recent laboratory demonstrations and thermal cycling remains a key constraint [112].

Passive strategies encode curvature directly into the material structure. Agharese and Okamura [113] thermally formed coated textiles into prescribed shapes, using heat‐set folds and local stiffness changes to define the path. Jitosho et al. [114] combined preformed geometry with Velcro locking and a high‐density PE line, allowing curvature to be maintained without continuous power. These approaches are lightweight and energy‐efficient, but their adaptability depends on fold and interface reversibility, fatigue resistance, and reprogrammability.

Overall, these material contributions involve trade‐offs among deformation, force, autonomy, and integration. Pneumatic films and anisotropic textiles provide reversible deformation and are compatible with everting bodies, but require pressure supply, reliable seams, and stable frictional interfaces. Magnetic composites enable wireless actuation and are attractive for confined or miniaturized systems, but require external field hardware and a compromise between magnetic loading and compliance. Shape‐memory and photothermal actuators integrate stimulus response with local deformation, yet thermal lag, energy use, and environmental compatibility remain major constraints. Preformed and locking structures require little holding power, but provide limited path adaptability and may rely on fatigue‐sensitive interfaces.

Among current strategies, pressure‐driven steering has the broadest experimental validation base, with repeated demonstrations across robot sizes and architectures, although long‐cycle reliability remains insufficiently characterized. Passive preforming and locking are technically accessible but remain application‐specific. Magnetic steering has been repeatedly validated in laboratory prototypes but still depends on specialized field systems. Thermally responsive approaches span a wider evidence range: SMA and photothermal phase‐change actuators remain largely proof‐of‐concept, whereas recently reported LCE‐integrated skins extend laboratory validation to millimeter‐scale, multibend steering. Across the literature, curvature, steering force, response time, energy demand, growth‐pressure penalty, and cycle‐life metrics are reported inconsistently, which limits rigorous cross‐platform comparison.

### 3.2 Stable Retraction

While steering governs deployment, retraction determines whether a vine robot can be recovered, reused, and removed safely. Retraction is governed by structural design and control, but its stability is strongly influenced by material properties. Thin everting bodies have low axial compressive stiffness and may fold laterally when the inversion force exceeds their resistance to buckling. This tendency becomes more severe with increasing curvature, unsupported length, and friction. Stable retraction therefore requires coordinated control of body stiffness, pressure‐supported geometry, interfacial friction, adhesion, and cyclic durability.

Coad et al. [32] established criteria distinguishing controlled inversion from unstable buckling in straight and curved robots. Their analysis linked retraction behavior to internal pressure, effective retracting length, body geometry, and friction. A tip‐mounted device reduced the effective retracting length to nearly zero and therefore limited the compressive load transmitted through the soft body. Although mainly a mechanical solution, it compensates directly for the low compressive stiffness of thin‐film bodies. Haggerty et al. [79] similarly localized steering and reeling near the tip. In this design, sliding resistance between the body material and the mechanism became critical: low friction reduces retrieval force, whereas excessive friction or wear can cause jamming and damage during repeated cycles.

Pressure‐supported material architectures provide a more integrated route. Wu and Mintchev [24] replaced a single everting tube with interconnected inflatable tubes (Figure 10D). Eversion occurred through 180° bending, allowing the bending point to translate with little volume change. The compliant tubes reduced the inversion force, while the pressurized tail and central connecting tube constrained lateral displacement. This combination suppressed bending moments and enabled buckle‐free retraction without a distal retraction device. Kim et al. [115] used a nested body containing a pressurized retraction channel. The channel acted as a compliant beam with pressure‐dependent bending stiffness and generated a restoring moment when lateral deflection began. Its performance therefore depends on channel geometry, wall compliance, seam integrity, pressure retention, and fatigue resistance.

Functional surfaces can stabilize retraction through environmental interaction. Qin et al. [26] integrated a microstructured adhesive film onto the robot surface. Interfacial adhesion counteracted radial forces that would otherwise displace the body from the supporting wall. During retrieval, the film was progressively peeled from the surface, enabling curved‐path retraction while limiting environmental damage. This strategy shifts stabilization from bulk stiffness to controlled adhesion and peel behavior. Its effectiveness, however, is sensitive to surface roughness, contamination, humidity, peel rate, and degradation of the microstructure.

Overall, material‐enabled retraction can reduce reliance on rigid tip hardware and distribute stabilization along the body. Pressure‐supported channels provide tunable restoring stiffness, low‐friction interfaces reduce tensile demand and wear, and adhesive skins can use environmental support without increasing bulk rigidity. These benefits involve clear trade‐offs. Greater stiffness or pressure may hinder eversion and increase stored energy; lower friction may reduce positional stability; and stronger adhesion may raise the peel force or fail on wet, dusty, or irregular surfaces. Repeated inversion also causes creasing, coating wear, seam fatigue, and leakage.

Stable retraction has been repeatedly demonstrated in controlled laboratory systems and is among the more developed functional capabilities of vine robots. However, most evidence remains platform‐specific. Long‐distance retrieval, standardized cycle‐life testing, and operation on contaminated, abrasive, or uncertain paths remain insufficiently validated.

### 3.3 Multifunctional Tip Design

In robotics more broadly, end‐effectors define the interface for tool use and environmental interaction [116], while soft grasping strategies show how compliant geometry can create stable but gentle contact [117]. In vine robots, the related engineering challenge is to maintain sensing, tool‐delivery, and payload functions at a distal region that moves relative to the everting wall. Here, multifunctional tip design is treated as an engineering integration challenge rather than a direct biological analogue. The central materials challenge arises from the continuous relative motion between the tip module and the robot wall. During eversion, the stored inner membrane is redirected at the growing front and becomes the deployed outer wall; during inversion, this process is reversed. A tip module must therefore remain localized relative to the moving front while accommodating repeated wall bending and rolling or sliding contact. It should minimize interfacial resistance, local stiffening, and stress concentrations that could disturb eversion or destabilize retraction.

Jeong et al. [25] addressed this problem using an outer cap, an internal retraction mechanism, and a rolling interlock. The interlock moved with the advancing or retracting tip, maintaining the payload at a fixed relative position (Figure 10E). Its load‐bearing components also transmitted sufficient tensile force to pull a 2.5 kg load. From a materials perspective, reliable operation requires a low‐friction rolling interface, adequate tensile strength, and sufficient local stiffness to prevent deformation under payload and retrieval forces. Excessive stiffness or surface friction would increase resistance at the eversion front, whereas insufficient strength or wear resistance could cause slippage, deformation, or failure during repeated retraction.

Suulker et al. [100] instead developed a soft cylindrical cap made from elastic textile or from inelastic fabric combined with rubber bands (Figure 10F). Here, attachment depends on friction between the cap and the outward‐moving body layer. Elastic fabrics provide conformability and distributed contact pressure, which improve fit and reduce rigid stress concentrations. However, viscoelastic relaxation may reduce gripping force over time. Inelastic textiles offer greater dimensional stability, while rubber bands supply recoverable tension. Their performance remains sensitive to fabric friction, elastic fatigue, surface contamination, and variations in body diameter.

Heap et al. [101] integrated a flexible pneumatic retraction element with an internal camera mount (Figure 10G). The soft pneumatic structure reduced mass by 40% relative to a motor‐driven device while providing comparable power. Its compliance also reduced geometric interference and protected the sensing unit inside the robot. Stable retraction, however, depends on pressure retention, wall flexibility, seam integrity, and cyclic resistance to creasing and leakage.

More recently, Yanez Trujillo et al. [118] developed a 7 mm vine robot with a magnetically retained tip mount that preserved a 3 mm working channel while integrating an RGB camera and LED illumination. The internal–external ring‐magnet configuration was designed to balance magnetic attachment force against the pressure and friction associated with eversion. The system achieved ±90° magnetic steering and was demonstrated in a bronchial phantom. This study provides stronger evidence for multifunctional distal integration because steering, visualization, and tool passage were combined within one compact tip module.

Overall, material‐based soft tip designs offer lower mass, greater conformity, and better compatibility with steering than rigid external mounts. Their limitations include lower positional precision, friction‐dependent retention, pressure sensitivity, and uncertain long‐term durability. Rolling‐interlock, pneumatic, and magnetic tip‐mount systems have been validated in laboratory prototypes, including recent integration of steering, visualization, and a working channel in a millimeter‐scale platform. Textile friction caps remain more limited proof‐of‐concept designs. Evidence for repeated payload retention, interface wear, leakage resistance, contamination tolerance, and long‐term multifunctional operation remains sparse.

### 3.4 Variable Stiffness

Across soft robotics, stiffness modulation is used to balance compliance during interaction with load‐bearing or force‐transmission requirements [119, 120]. In vine robots, the analogous conflict is between low bending resistance during growth and high load‐bearing capacity after deployment. Natural vines resolve this conflict through spatially graded tissue maturation. Compliant apical tissues facilitate exploration and twining, whereas lignified proximal tissues provide structural support. Vine robots seek a functional analogue by changing the mechanical state of selected body regions without excessively restricting tip growth.

Do et al. [31] retained a compliant LDPE pressure body and added distributed jamming pouches containing patterned polyester‐fiber layers (Figure 11A). The low bending stiffness of LDPE preserved foldability and eversion, whereas the polyester layers provided high in‐plane strength and frictional interfaces. When a pressure difference compacted the layers, interlayer friction suppressed sliding and increased the local bending stiffness. Releasing the pressure difference restored layer mobility and segment compliance. Magnetically controlled ball valves selected the segments to be stiffened. At a body pressure of 6.9 kPa, the force required to produce a 10 mm transverse displacement increased from 1.07 N in the unjammed state to 6.68 N in the jammed state, corresponding to the reported 524% increase in transverse load resistance. This improvement enhanced shape retention, self‐support, and directional stability while allowing the remaining body to stay compliant. A subsequent design used a thinner layer‐jamming architecture driven directly by the pressure difference between the positively pressurized robot body and the atmosphere [28, 121]. This configuration reduced additional bulk and enabled stiffness switching within seconds. Triangular‐wave interfaces between adjacent units improved load transfer and reduced hinge‐like deformation, thereby enhancing upright stability. Feteih et al. [122] introduced a third pneumatic route by integrating axially stacked pouches into the robot skin. Inflating the pouches generated axial tension that suppressed wrinkling and increased transverse load resistance while allowing continued growth. In an 18 mm‐diameter platform, the mechanism produced a reported 680% increase in stiffness. Unlike layer jamming, the effect arises from pressure‐generated membrane tension rather than interlayer friction, and unlike LMPAs, it avoids thermal phase change. Its main penalties are the additional skin layers, high local pressure, seam loading, and added eversion resistance.

**FIGURE 11:**
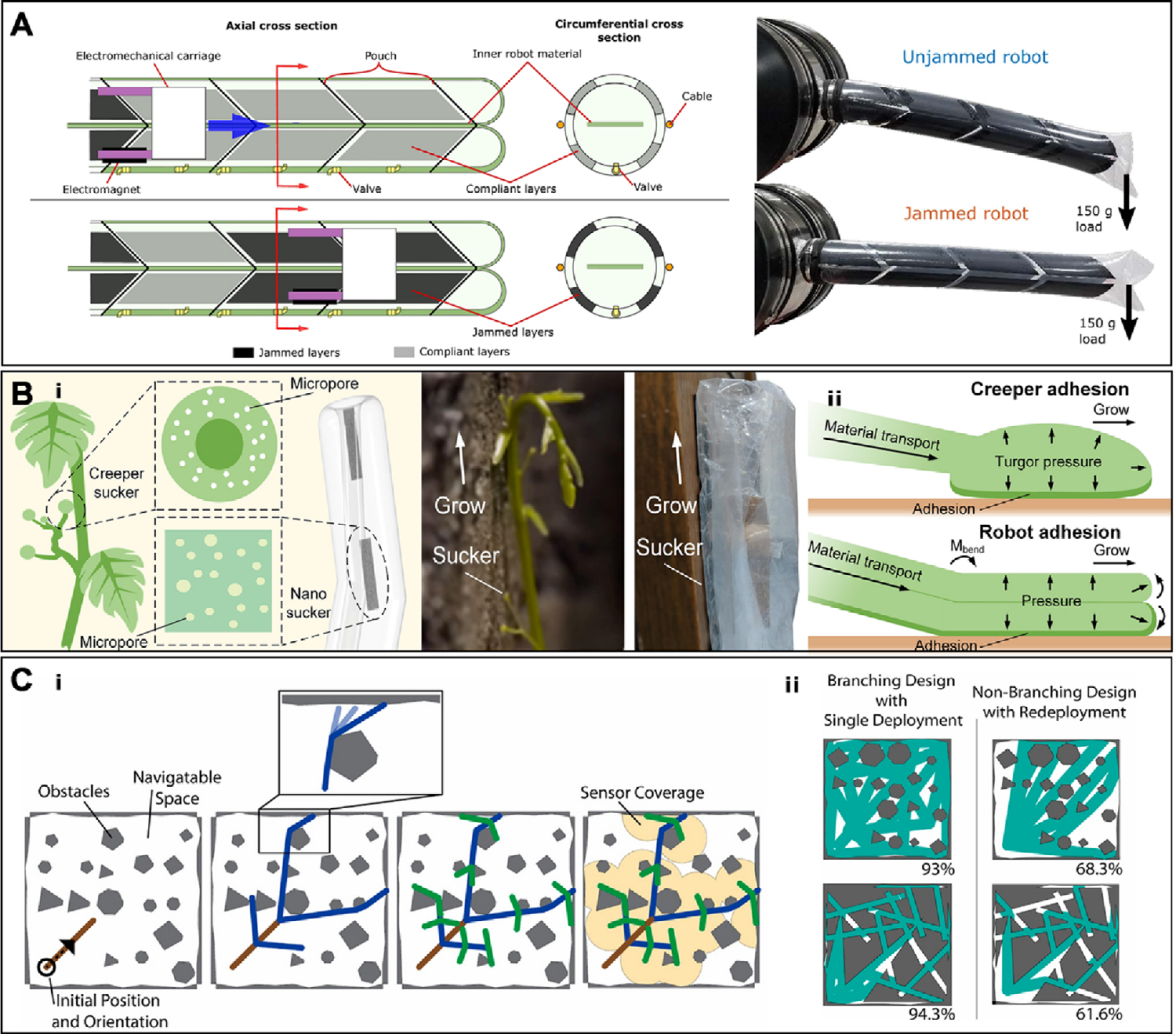
Material‐enabled variable stiffness, climbing and attachment, and branching. (A) Distributed layer‐jamming body and load‐bearing response in unjammed and jammed states. (B) (i) Creeper‐inspired adhesive film with micropores and nanosuckers, and (ii) growth‐assisted climbing. (C) (i) Branched deployment strategies and (ii) resulting coverage in grid and cave environments. (A) Reproduced with permission [31]. Copyright 2020, IEEE. (B) Reproduced with permission [26]. Copyright 2025, AAAS. (C) Reproduced under the terms of the CC BY 4.0 license [27]. Copyright 2022, The Authors, published by Frontiers.

Low‐melting‐point alloys provide a second and mechanistically distinct route to variable stiffness. As discussed in Section 2.2.1, Venkiteswaran et al. [10] encapsulated Rose's metal within a silicone tube and used resistive heating to control its phase state. Selective melting produced a compliant distal region that could be deformed and magnetically guided, whereas resolidification converted the proximal region into a load‐bearing structure. The measured stiffness changed from 0.016 N m^−^^1^ in the liquid state to 1.304 N m^−^^1^ in the solid state, corresponding to a stiffness ratio of approximately 83. Mao et al. [81] extended this principle using Cerrolow‐117 in a follow‐the‐leader architecture. The Guider and Follower alternated between compliant and rigid states, allowing one component to define the trajectory while the other reproduced and mechanically stabilized it. More recent work has further demonstrated localized, reversible phase‐change stiffening and actuation in steerable eversion tip‐growing robots [82]. These systems qualify as variable‐stiffness robots because their mechanical state can be actively and reversibly changed. However, unlike an auxiliary post‐deployment stiffening module, their phase transition is intrinsically coupled to growth, steering, and path retention.

Layer jamming and LMPAs therefore reproduce the spatial maturation of natural vines through different material mechanisms. Layer jamming changes the mobility of internal interfaces while leaving the constituent materials in the same physical state. It is lightweight, locally addressable, reversible, and compatible with the pneumatic supply already required for eversion. It also avoids thermal exposure and can stiffen selected, already‐deployed segments without changing the mechanics of the growing tip. However, sufficient pressure difference must be maintained, and the achievable stiffness depends strongly on frictional contact. Leakage, contamination, wear, wrinkling, and layer misalignment can reduce performance. Additional pouches, seams, valves, and patterned interfaces also increase body thickness, fabrication complexity, storage volume, and eversion resistance.

By contrast, LMPAs change the intrinsic mechanical state of the load‐bearing material. Solidification provides a larger stiffness contrast and solid‐like force transmission, and the solidified segment can retain its shape without maintaining a pneumatic jamming pressure. The alloy can also be confined within a relatively compact channel. However, continued growth or reshaping requires the distal region to remain molten or to be reheated. Switching speed is consequently governed by heat transfer, latent heat, heater power, alloy volume, and environmental cooling. The high density of metallic alloys increases body mass, while imperfect encapsulation creates risks of leakage and interfacial delamination. Pb‐ and/or Cd‐containing LMPA formulations, including Rose's metal and Cerrolow‐117, also raise toxicity and biomedical‐safety concerns. Thermal exposure to surrounding tissues or temperature‐sensitive environments must therefore be carefully controlled.

The preferred mechanism consequently depends on the required balance among growth continuity, stiffness contrast, load capacity, response time, mass, energy consumption, and environmental compatibility. Pneumatic layer jamming is better suited to rapidly and selectively reinforcing an otherwise compliant everting body. Phase‐change alloys are more suitable when high stiffness contrast, persistent path retention, and mechanically stable proximal regions justify the added thermal and encapsulation requirements. The newer pneumatic‐pouch route occupies an intermediate position by increasing structural support through membrane tension without jamming interfaces or thermal phase change, but it adds pressurized layers and seam loads to the growing skin. Current systems therefore represent distinct trade‐off profiles rather than a converged variable‐stiffness solution.

Available variable‐stiffness systems remain laboratory prototypes rather than deployment‐ready technologies. Reported studies emphasize stiffness ratios or selected force–displacement metrics, whereas absolute bending and torsional stiffness, specific stiffness relative to added mass, switching time, energy consumption, pressure or thermal losses, spatial resolution, and cyclic durability are not reported consistently. Evidence under leakage, contamination, abrasion, humidity, long‐term interfacial wear, heating/cooling nonuniformity, phase‐transition fatigue, encapsulation damage, and orientation‐ or temperature‐dependent environments is also limited. This uneven evidence base makes direct comparison among layer‐jamming, pneumatic‐pouch, and LMPA systems difficult and leaves long‐duration load‐bearing reliability insufficiently established.

### 3.5 Climbing and Attachment

Climbing shifts vine‐robot interaction from the tip to the body–environment interface, allowing the deployed body to obtain mechanical support on vertical or irregular surfaces [123, 124, 125, 126]. Existing strategies can be grouped into shape locking, multi‐stem twining, and growth–adhesion coupling [127]. Although structurally distinct, their performance is governed by a common set of material properties, including compliance, friction, tensile strength, surface microstructure, and interfacial durability.

Wang et al. [128] developed a variable‐length shape‐locking vine robot inspired by the stiff proximal and compliant distal regions of natural vines. Two tendons controlled bending, while two inextensible pneumatic locking elements extended along the sides of the everting body. When pressurized, each element expanded against a guide tube. The resulting normal force, combined with the high friction of a silicone‐coated interface, suppressed relative sliding and fixed the local curvature. The locked proximal region therefore supported the deployed path, whereas the unlocked distal region remained compliant and steerable. This spatial control of friction increased the reachable workspace and enabled the robot to maintain trajectories around obstacles. The locking performance depends on chamber compliance, inflation pressure, coating friction, and wear resistance. However, additional layers increase body thickness and eversion resistance, while elastomeric hysteresis and repeated sliding may reduce repeatability.

Multi‐stem twining generates attachment through distributed wrapping rather than local shape fixation. Shan et al. [129] constructed a woven gripper containing eight longitudinal fibers constrained by transverse fibers. Counter‐rotation of the inner and outer fiber groups produced multilayer wrapping around the target. The flexible textile network accommodated geometric variations, whereas fiber tensile strength and interfacial friction converted wrapping into load‐bearing confinement. The reported static and dynamic peak load ratios reached 151: 1 and above 435: 1, respectively. Compared with single‐stem twining, the multi‐stem architecture enlarges the contact area, redistributes stress, and reduces sensitivity to local slip. Nevertheless, its performance depends on fiber abrasion resistance, crossover friction, and fatigue under repeated twisting. It also requires rotary actuation and was demonstrated as a gripper rather than as an integrated growing‐climbing robot.

Qin et al. [26] coupled growth directly with surface adhesion by integrating a compliant microstructured film onto the robot body (Figure 11B). During eversion, the film turned outward and contacted the substrate. Its low effective modulus promoted conformal contact, while microcavities generated negative pressure and close interfacial contact enhanced van der Waals interactions. Adhesive stresses reached approximately 10 kPa on smooth surfaces and 7 kPa on rough surfaces. Because adhesion formed progressively during growth, the attached regions provided distributed support without a separate anchoring cycle. However, performance is sensitive to roughness, dust, moisture, surface chemistry, and damage to the microstructures during repeated eversion and peeling.

Overall, shape locking offers controllable path support but introduces pneumatic complexity, added thickness, and frictional wear. Twining provides strong, shape‐adaptive attachment to discrete objects, but current evidence is largely transferable from adjacent bioinspired gripper designs rather than integrated growing‐climbing platforms. Growth‐adhesion coupling is the most direct route to continuous surface climbing, although its reliability is strongly environment‐dependent. The evidence base therefore remains limited but growing. Laboratory demonstrations establish feasibility, whereas systematic data on cyclic wear, contamination tolerance, load retention, substrate diversity, and long‐duration climbing remain sparse.

### 3.6 Branching Design

Natural climbing plants combine regulated shoot branching with diverse attachment adaptations to expand access to surrounding space [130, 131]. Light is also a major regulator of bud outgrowth and branching [132]. In vine robots, branching similarly expands morphology to improve spatial coverage and reduce dependence on a single growth trajectory. This capability is valuable in uncertain environments, where an incorrect path may otherwise require complete retraction and redeployment. Branching, however, is not determined by geometry alone. It requires a material system that can form sealed junctions, withstand pressure‐induced membrane loads, and remain compliant enough for compact storage and eversion.

Glick et al. [27] combined a parameterized branching architecture with particle swarm optimization. Branching depth, branch length, branch angle, and branch number were optimized for representative grid and cave maps, then evaluated in unknown environments of the same type (Figure 11C). The branching robots achieved 93% coverage in grid environments and 94.3% in cave environments, compared with 68.3% and 61.6%, respectively, for non‐branching robots. These gains arose from morphological redundancy, as multiple branches distributed exploration across several directions and reduced the consequence of selecting an unfavorable path.

The hardware was fabricated from TPU film by laser welding. TPU flexibility allowed the branched body to be folded, stored, and everted, while its tensile and tear resistance supported membrane loading near junctions. Thermoplastic weldability also enabled sealed bifurcations that would be difficult to produce reliably with less weldable films. However, branch junctions create abrupt geometric and stiffness changes. Weld width, heat‐affected regions, film thickness, and seam quality therefore govern leakage, stress concentration, and burst resistance. Increasing thickness or using a stiffer TPU grade improves pressure tolerance but raises bending resistance, storage volume, and eversion pressure. Pressure and stored material must also be distributed among branches, complicating uniform deployment and independent control.

More recently, Satake et al. [133] demonstrated on‐demand branch formation using localized heat cutting rather than a fully predefined branched body. A movable unit clamped the pressurized LDPE trunk with compliant silicone‐rubber sealing elements, heated the film with a nichrome wire, and allowed a preloaded branch tube to emerge through the newly formed opening. This approach changes the material problem from fabricating a fixed welded junction to controlling local thermal weakening while maintaining pressure sealing around the cut. Branching time depended on internal pressure and branch diameter, and branches could be created at selected positions along the trunk. The demonstration expands branching beyond predefined morphology, but it also introduces new constraints in thermal damage, sealing reliability, branch‐tube routing, and recovery.

Overall, branching remains the least extensively validated capability in this section. Predesigned TPU architectures demonstrate that sealed bifurcations can increase spatial coverage, whereas heat‐cutting systems show that branches can also be formed on demand along an already deployed trunk. These routes impose different material constraints: welded branches depend on junction geometry, heat‐affected seams, and stored‐material distribution, while on‐demand branching depends on localized thermal weakening, temporary sealing, and controlled rupture of the trunk film. Both approaches add pneumatic and routing complexity and make recovery more difficult. The present evidence is therefore best described as proof‐of‐concept laboratory validation rather than a general branching capability.

### 3.7 Comparative Discussion of Functional Capabilities

The six capabilities reviewed above do not represent a single class of vine‐inspired actuation. Rather, they describe functional requirements that arise once tip growth is established. Steering, variable stiffness, climbing/attachment, and branching retain relatively direct biological analogies, whereas retraction and multifunctional tip integration mainly address engineering requirements for recoverability and task execution. Across all six functions, materials contribute by redistributing compliance, stiffness, friction, adhesion, magnetic response, or phase state while preserving the low resistance required for eversion or tip‐localized growth.

The experimental evidence is uneven. Controlled steering has the broadest validation base, spanning pneumatic, passive, magnetic, SMA, photothermal, and LCE‐based approaches across multiple scales. Stable retraction is also comparatively developed, with localized mechanisms and pressure‐supported channels repeatedly demonstrated, including recent millimeter‐scale fully soft systems. Multifunctional tip integration has progressed from frictional caps and rolling interlocks to compact modules combining magnetic steering, imaging, and tool passage, but durability data remain platform‐specific. Variable stiffness is supported by layer‐jamming, pneumatic‐pouch, and phase‐change systems, which trade switching speed and mass against stiffness contrast, pressure demand, and thermal burden. Climbing and attachment remain more substrate‐ and application‐dependent; direct growth‐coupled adhesion is supported by fewer platforms than the adjacent twining and locking concepts. Branching has the smallest evidence base, although recent work now distinguishes predefined branched morphologies from on‐demand branch formation. Taken together, present vine‐robot functionality is enabled less by any single “smart” material than by the coordinated design of material properties, interfaces, fabrication methods, and body architecture.

## 4 Multifunctional Applications

Applications provide the clearest test of whether material choices made for growth and functional capabilities remain effective under task‐specific constraints. Accordingly, this section is organized by application demand rather than by robot architecture. Medical use prioritizes low interaction force, miniaturization, and biocompatibility; navigation and exploration emphasize damage tolerance and environmental compatibility; transport and target‐side operations require reliable pressure‐ and medium‐compatible pathways together with payload stability; and deployable or reconfigurable structures require functional continuity across mechanically dissimilar interfaces. This section highlights representative advances in four major domains (Figure 12).

**FIGURE 12:**
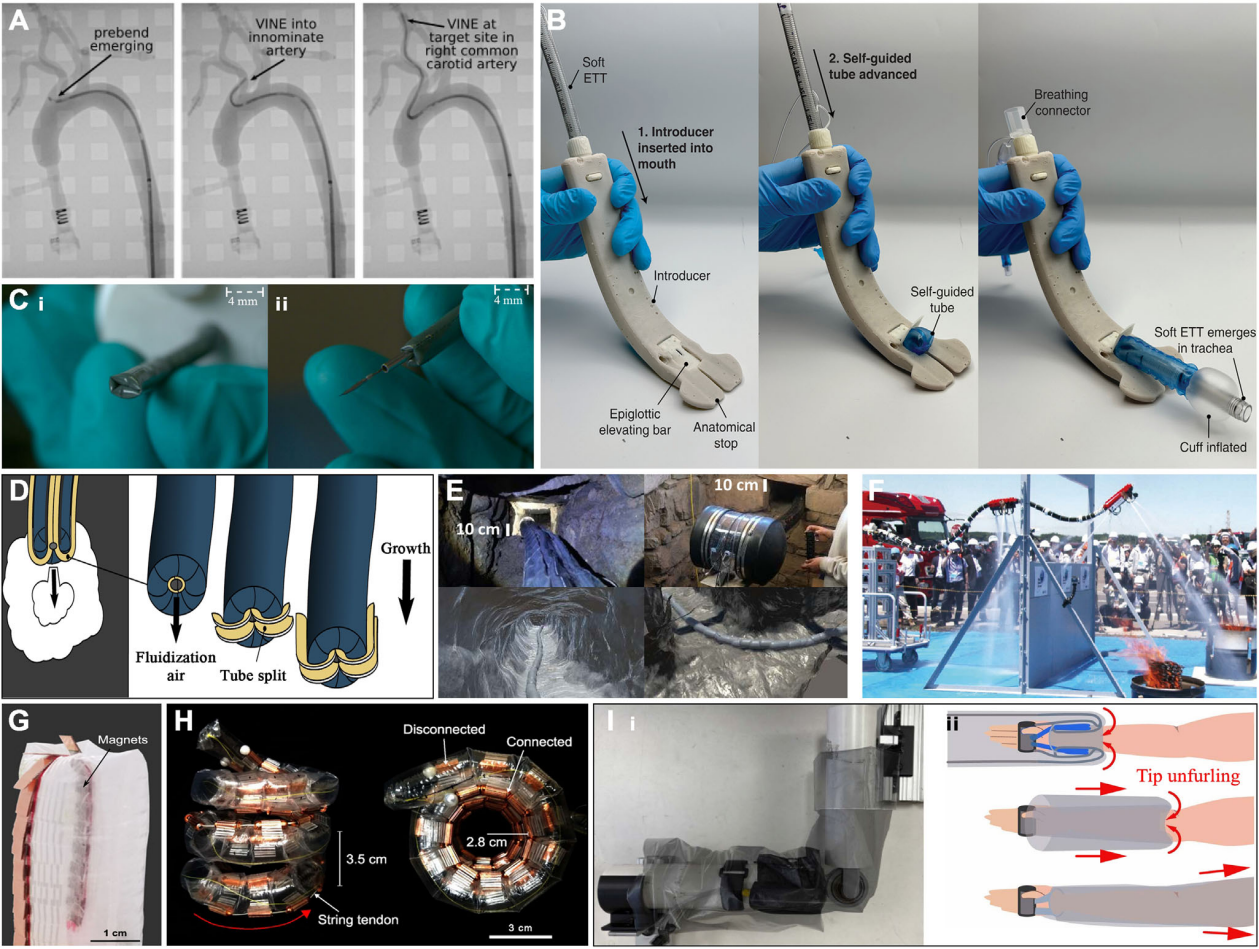
Representative application‐oriented vine robots and closely related long‐reach soft systems. (A) Endovascular VINE catheter. (B) Self‐guided robotic intubation. (C) Magnetic‐fluid‐driven biopsy. (D) Granular‐fluidization‐assisted burrowing. (E) Archaeological field exploration. (F) Dragon Firefighter. (G) Growable frequency‐reconfigurable antenna. (H) SuBB‐enabled reconfigurable electromagnetic structure. (I) Self‐wearing adaptive garment: (i) prototype and (ii) working principle. (A) Reproduced with permission [134]. Copyright 2021, IEEE. (B) Reproduced with permission [135]. Copyright 2025, AAAS. (C) Reproduced with permission [109]. Copyright 2025, Wiley‐VCH GmbH. (D) Reproduced with permission [13]. Copyright 2018, IEEE. (E) Reproduced with permission [11]. Copyright 2020, IEEE. (F) Reproduced under the terms of the Creative Commons Attribution 4.0 International License [15]. Copyright 2023, The Authors. (G) Reproduced with permission [14]. Copyright 2018, IEEE. (H) Reproduced with permission [136]. Copyright 2020, IEEE. (I) Reproduced with permission [29]. Copyright 2026, IEEE. SuBBs, subwavelength‐scale building blocks; VINE, vascular internal navigation by extension.

### 4.1 Medical Diagnosis and Intervention

Minimally invasive procedures impose several coupled requirements on robotic devices. They must pass through narrow, tortuous, and fragile lumens, including blood vessels, airways, and the gastrointestinal tract, while limiting tissue interaction forces [137, 138, 139]. They must also negotiate highly curved or branching anatomies and, in many cases, deliver sensing or interventional tools to the target site. These requirements translate directly into material constraints. The body should have sufficiently low bending resistance for atraumatic growth, while retaining enough tensile strength and pressure integrity to prevent excessive deformation or failure. Functional materials used for steering must generate controllable forces or torques without substantially compromising body compliance. Tip eversion is advantageous in this setting because it limits sliding of the deployed body, but this advantage is retained only when the membrane, seams, and functional layers remain compliant and mechanically reliable at the relevant scale.

For atraumatic access, the primary material requirement is a compliant but pressure‐retaining body. Slade et al. [12] demonstrated that tip eversion substantially reduces relative sliding during catheter advancement. Combined with preformed trajectories and pressure‐dependent structural support, the catheter navigated complex paths in brain‐tissue phantoms with a lower risk of puncture. Li et al. [134] further illustrated how structural materials can be tailored to intravascular requirements. Their vascular internal navigation by extension (VINE) catheter used a 0.1 mm TPU‐coated nylon tube (Figure 12A). The coated textile combined low bending resistance for eversion with reinforcement and pressure retention during navigation. A pre‐curved geometry with a radius of 20 mm further matched vascular structures such as the aortic arch. In the reported test conditions, the maximum wall stress was 165.9–234.3 kPa, approximately one‐sixth to one‐eighth of that produced by conventional catheters (∼1440 kPa), while navigation over a 0°–180° range was achieved. The reported procedure time was also approximately one‐tenth of that required by the comparator. These results illustrate a key material–geometry trade‐off: reducing bending resistance facilitates low‐trauma advancement, whereas pressure integrity and local reinforcement are still required to preserve the intended trajectory.

A similar requirement arises in airway access, where the device must conform to highly curved anatomy without imposing large lateral forces. Haggerty et al. [135] developed a self‐guided intubation system based on a soft, tip‐extending TPU overtube that everts while advancing a compliant endotracheal tube (Figure 12B). The overtube can be formed into a sigmoid geometry approximating the airway, and tip extension minimizes relative motion against surrounding tissue. Here, material compliance and preformed geometry work together rather than independently: compliance reduces contact forces, whereas prescribed curvature biases the growing tip toward the desired anatomical path. Miniaturization adds another constraint because decreasing robot diameter leaves less space for steering elements, sensors, and working channels. Oyejide et al. [20] highlighted this coupling among miniaturization, material compliance, steering, sensing, and operator feedback in soft growing robots for minimally invasive surgery.

Once the robot reaches a branching anatomy, directional controllability becomes a second material requirement. Magnetic materials are attractive because they can generate steering forces without bulky onboard actuators [140, 141]. Davy et al. [98] coated the robot body with a silicone layer containing NdFeB nanoparticles. The elastomeric matrix maintained flexibility, while the magnetic particles provided sufficient remanent magnetization for remote steering. This combination enabled an approximately 8 mm‐scale robot to selectively enter branches in bronchial models under coupled pneumatic and magnetic actuation. The material design nevertheless introduces a trade‐off: increasing magnetic loading can enhance magnetic response, but excessive functionalization can increase stiffness and place greater mechanical demands on the coating–substrate interface during repeated eversion.

Davy et al. [109] subsequently adopted a different strategy by using a magnetic fluid within the growing robot (Figure 12C). Incorporating the magnetic phase into the working fluid avoided repeated deformation of a particle‐filled surface coating and increased the available magnetic volume fraction. The same fluidic system also supplied pressure for growth and distal intervention. With a miniature biopsy needle integrated at the tip, the robot generated a tissue penetration force of 1.26 N, demonstrating that a compliant navigation platform can transmit useful forces after reaching the target. This approach shifts the dominant reliability requirement from coating adhesion toward fluid containment, sealing, and stable magnetic‐fluid behavior. Phase‐changing materials provide another route to reconcile navigation compliance with positional stability. Mao et al. [81] used magnetically guided phase‐transition elements to alternate between compliant and rigid states, enabling follow‐the‐leader advancement with limited mechanical interaction. Such designs show that changes in intrinsic material state can support both steering and structural stabilization, although thermal exposure and material composition remain important constraints for medical use.

These studies reveal a common materials principle for minimally invasive vine robots: the distal region should remain sufficiently compliant and steerable for safe navigation, whereas the deployed body and functional tip must provide enough mechanical stability to retain shape and transmit useful forces. No single material currently satisfies all of these requirements. Thin polymer films reduce bending resistance but remain vulnerable to puncture and sealing defects. Coated textiles improve strength and durability but can increase thickness and eversion resistance. Magnetic particle composites add remote steering but introduce stiffness and interfacial‐fatigue trade‐offs, whereas magnetic fluids reduce coating‐related failure at the cost of stricter containment requirements. Mechanically anisotropic magnetic structures, although demonstrated in related minimally invasive devices rather than vine robots, further show how directional stiffness can suppress undesired bending and twisting under misaligned magnetic fields. The present evidence therefore supports a co‐design problem rather than a single‐property optimization: compliance, pressure integrity, functional‐material response, and interfacial reliability must be maintained simultaneously as the device is miniaturized. Clinical maturity remains limited. Most systems have been validated in phantoms or controlled laboratory settings, while biocompatibility, hemocompatibility, extractables and leachables, particle or fluid leakage, sterilization stability, thermal exposure, puncture and burst resistance, and cyclic seam or coating durability have not yet been demonstrated together under clinically representative conditions.

### 4.2 Navigation and Exploration

Navigation and exploration place vine robots in collapsed structures, subsurface soil, archaeological passages, and fragile underwater environments. All are confined and geometrically uncertain, but the dominant material load differs by environment. Rubble introduces puncture, cutting, and abrasion; granular media generate distributed resistance that can arrest penetration; and underwater operation adds buoyancy, sealing, biofouling, and low‐disturbance requirements [142]. The material problem therefore shifts from simple compliance to environment‐specific protection. The body must remain foldable and pressure‐retaining while local actuators, coatings, reinforcements, and sensors are added without raising eversion resistance everywhere. Tip growth remains advantageous because the deployed body undergoes little translation relative to the surroundings [143].

In subsurface navigation, the key materials challenge is to overcome particle resistance without increasing body stiffness. Ozkan‐Aydin et al. [144] integrated two sPAMs along a thin‐film everting body. Alternating inflation generated lateral tip oscillation, while the compliant film retained the low bending resistance required for eversion. This localized deformation enlarged the explored region and increased the penetration depth to more than three times that achieved without oscillation. The design demonstrates that spatially confined soft actuation can improve penetration without requiring a uniformly thicker or stiffer body. Its limitation is added pneumatic complexity: additional chambers and seams increase pressure demand and introduce potential leakage and fatigue sites. Naclerio et al. [13] instead reduced the mechanical resistance of the surrounding medium. Their underground growing robot used an LDPE outer tube as the compliant pressure body and an inner nylon tube as a dedicated airflow channel (Figure 12D). Air delivered at the tip locally fluidized packed particles and reduced the penetration force by approximately one order of magnitude compared with direct movement of a similarly sized object. This allowed the LDPE body to remain thin and foldable despite its limited load‐bearing capacity. The system illustrates a broader material–environment principle: when a soft membrane cannot tolerate high external loads, the environment can be temporarily modified to reduce them.

Collapsed structures impose a different trade‐off. The body must bend through irregular voids while resisting puncture, cutting, abrasion, and seam failure. McFarland et al. [145] evaluated the Soft Pathfinding Robotic Observation Unit in simulated urban‐search‐and‐rescue environments containing gravel, reinforcement bars, and narrow openings. Although the robot accessed regions unavailable to humans and conventional platforms, the tests exposed the vulnerability of soft body materials to sharp debris. These results show that low bending stiffness alone is insufficient; tear resistance, abrasion tolerance, seam strength, and repairability are also essential. Coad et al. [11] addressed these requirements using TPU‐coated ripstop nylon. The woven nylon reinforcement increased tensile, tear, and abrasion resistance, whereas the TPU coating provided a pressure‐retaining and sealable surface. The composite remained sufficiently foldable for storage and eversion. The robot crossed sand, passed through a 4.5 cm opening, climbed stairs, and navigated around obstacles in the RoboSoft 2018 competition. It later grew 5–6 m through narrow passages at the Chavin archaeological site and negotiated rock obstacles, a 90° turn, and a vertical ascent (Figure 12E). In these rough‐field demonstrations, the coated textile provided a larger damage‐tolerance margin than unsupported ultrathin films. The gain in durability, however, increased wall thickness, areal mass, and bending resistance. Coating–fabric interfaces and welded seams also remained potential sites for delamination, leakage, and cyclic fatigue.

Fragile underwater environments require mechanical integrity with low ecological disturbance. Luong et al. [67] developed an underwater everting robot using silicone‐coated ripstop nylon. The textile reinforcement supported pressurization and environmental contact, while the compliant silicone coating maintained sealing and flexibility in water. Ambient water, rather than compressed air, was used as the working fluid. This reduced density mismatch, enabled near‐neutral buoyancy, and lowered friction and disturbance around coral reefs. In this case, the working fluid contributed to both actuation and buoyancy control. The remaining material liabilities are coating delamination, water ingress, saltwater aging, biofouling, and repeated hydrodynamic loading.

Obstacle‐aided navigation shows that compliant growing bodies can deliberately exploit environmental contact [146]. Sensing integration introduces a different materials challenge because local reinforcement can hinder folding and eversion [147]. Gruebele et al. [148] used LDPE as the lightweight structural body and polyimide‐based flexible printed circuit boards for distributed IMUs and temperature and humidity sensors. Vytaflex 60 polyurethane was added around vulnerable electronic regions as a mechanical bend limiter. This spatially selective protection reduced excessive local curvature without stiffening the entire body. However, circuit boards, encapsulants, and interconnects create discontinuities in thickness and modulus that can increase eversion resistance, concentrate stress, and fatigue during repeated deployment. Contact‐guided navigation similarly depends on predictable bending, friction, and surface durability, linking path repeatability to body material properties [149]. The key integration principle is therefore selective rather than uniform reinforcement: protection should be placed where fragile components require it while the remaining body preserves low bending resistance.

Across navigation and exploration, material selection is dominated by the balance between low deployment resistance and damage tolerance. Thin LDPE minimizes bending and eversion resistance in relatively controlled or low‐load conditions, whereas coated ripstop textiles provide greater pressure capacity and damage tolerance for rubble, archaeological, and underwater deployment at the cost of higher mass and bending resistance. Local soft actuators can improve penetration while preserving global compliance, and flexible electronics benefit from spatially selective protection rather than uniform encapsulation. The evidence spans laboratory soil, simulated debris, competition courses, archaeological field deployment, and underwater tests, making this one of the more environmentally diverse application groups. Direct comparison nevertheless remains difficult because studies use different media, robot scales, exposure durations, and success criteria. Cyclic abrasion and puncture, tear propagation, seam and coating fatigue, pressure leakage, environmental aging, contamination tolerance, sensor‐interconnect durability, and field repairability are rarely evaluated together. The unresolved materials problem is therefore spatially heterogeneous protection: reinforcement and encapsulation are needed where damage or functional discontinuities occur, but uniform thickening directly penalizes folding and eversion.

### 4.3 Transport and Operational Tasks

Transport and target‐side operations require more than access to a remote location. In firefighting, nuclear decontamination, and environmental sampling, the system must carry a fluid, sensor, or tool through a narrow or hazardous route and then maintain sufficient positional stability to act at the target. The material problem is therefore layered: a pressure‐ and medium‐compatible lumen is needed for transport, a compliant outer body is needed for access, and the distal structure must resist buckling and reaction loads. Environmental exposures such as water, chemicals, heat, radiation, and outdoor aging add a second set of constraints. Reported vine robots, together with closely related long‐reach soft hose systems, illustrate how these functions can be distributed among separate material layers and terminal modules.

Firefighting illustrates the transport–stability conflict, although the Dragon Firefighter is a related flying‐hose continuum robot rather than a tip‐growing vine robot [15]. Its inclusion is useful because it exposes material constraints that any long‐reach fluid‐delivery soft system must address. Yamauchi et al. [15] developed the 4 m‐long system using an inner polypropylene water‐supply hose and an outer polypropylene corrugated tube (Figure 12F). The inner hose provided a continuous pressure‐retaining flow path, whereas the corrugated tube accommodated cables and reduced local buckling while maintaining low bending stiffness. Distributed nozzle units converted the transported water into reaction thrust for levitation and steering, so water served as both the delivered medium and the actuation source. The corrugated geometry enabled long‐reach support without a rigid arm, but heat exposure caused detrimental plastic deformation of the polypropylene tube and altered the body mechanics. Practical systems will therefore require less thermosensitive polymers, thermal shielding, or multilayer structures that retain flexibility while limiting heat‐induced creep.

Nuclear decontamination imposes different constraints. The robot must traverse pipes, enclosed vessels, and T‐junctions while carrying sensors and delivering liquids, aerosols, or immobilizing foams. Its body must be foldable and tear‐resistant, whereas seams and barrier layers must preserve pressure and prevent leakage of contaminated fluids. Al‐Dubooni et al. [30] fabricated the everting body from sewn ripstop nylon strips and sealed the seam with heat‐pressed vinyl. The woven nylon provided flexibility, durability, and resistance to tear propagation, while the vinyl supplied the pressure‐retaining barrier required for pneumatic growth. A three‐degree‐of‐freedom continuum mechanism at the tip provided directional control and a rigid mount for a camera and sprayer. This hybrid design combined compliant access with stable target‐side spraying and achieved a success rate above 95% in controlled tests. Nevertheless, the rigid tip increased local diameter and stiffness, limiting access to smaller pipes. Resistance of the nylon, vinyl seal, sewn interfaces, and printed tip components to radiation, aggressive chemicals, and repeated decontamination remains unverified, so task success in controlled tests should not be equated with material qualification for nuclear environments.

Airborne sampling shifts the priority toward low mass, cantilever stability, and reliable retraction. Wu and Mintchev [24] developed a multi‐tube robot from 50 µm heat‐welded polyethylene tubes connected with acrylic adhesive tape. The low areal mass of the thin PE body enabled compact storage, while the interconnected geometry maintained alignment of the internal tail and reduced retraction‐induced buckling. The 2 m body weighed approximately 40 g and could retract from cantilevered configurations exceeding 1 m, supporting deployment from a drone into inner tree canopies as a potential route for localized environmental DNA sampling. The demonstrator still used pneumatic inflation; the material architecture removed the pressurized root chamber and tip‐mounted retraction device rather than eliminating the air supply. Strong curvature or external pinching increased capstan friction and could cause jamming. Accordingly, this work provides evidence for lightweight aerial deployment and retraction, not yet for an end‐to‐end environmental DNA sampling workflow. The material bottlenecks remain interfacial friction, bond durability, and puncture resistance at very low areal mass.

Across transport and operation tasks, task completion depends on functional separation among the transport lumen, compliant body, and terminal module. The lumen must provide pressure integrity and chemical compatibility with the delivered medium; the body must remain deployable while resisting buckling, puncture, and environmental degradation; and the tip must supply task‐level stiffness without becoming excessively large or heavy. The Dragon Firefighter demonstrates large‐scale remote extinguishing in a related continuum hose platform; the nuclear system has been evaluated in controlled spraying tasks; and the aerial system primarily validates deployment and retraction. No common material stack has yet been qualified across the thermal, chemical, radiation, outdoor, and retrieval demands represented by these tasks. The dominant unresolved issues are therefore medium compatibility, environmental aging, seam or adhesive fatigue, local buckling, tip‐size penalties, and reliable recovery after operation.

### 4.4 Deployable and Reconfigurable Structures

Deployable and reconfigurable structures differ from the previous three domains because the grown body is not only an access pathway; it becomes part of the final functional structure. This is attractive when a device must be stored compactly, routed through constrained environments, and then deployed or reshaped at the target site. Electromagnetic structures and wearable systems illustrate this application class. Their common material challenge is mechanical incompatibility: the carrier must be lightweight, foldable, pressure‐retaining, and compliant, whereas conductive or load‐transmitting elements must preserve function during inversion, bending, or repeated shape change. Interfaces between these mechanically dissimilar components therefore become primary design variables. In emergency communication, conventional reconfigurable antennas based on hinges or dielectric tuning offer limited shape change and often retain rigid elements [150, 151]. Vine robots instead use growth to establish the structure and body deformation to modify their geometry.

Blumenschein et al. [14] combined a thin LDPE body with copper foil and a steel backplane to create a growable, frequency‐reconfigurable antenna (Figure 12G). The low areal mass and foldability of LDPE enabled compact storage and pressure‐driven eversion. During growth, copper segments were mechanically disconnected at the tip, changing the effective electrical length without stretching a continuous conductor and producing a tuning range of 0.4–2 GHz. The compliant body could also form helical or multi‐element configurations and use trees or rubble as external support. The material architecture therefore separated large deformation in the polymer carrier from the lower admissible strain of the metal conductor.

Gan et al. [136] addressed post‐deployment reconfiguration using an inflatable LDPE tube and surface‐mounted copper subwavelength‐scale building blocks (SuBBs). The LDPE skeleton provided low bending resistance and reversible three‐dimensional deformation (Figure 12H). Instead of forcing a continuous metal trace to sustain large strain, the relatively rigid copper blocks formed conductive pathways through controlled joint contact. This architecture assembled helical electromagnetic structures and demonstrated functional antenna operation at 1.5 GHz. Conductivity remained stable over more than 10 reconfiguration cycles. The design again decouples carrier deformation from conductor strain, but transfers reliability to contact resistance, attachment durability, and pressure retention.

Recent work shows that the same principle can extend beyond electromagnetic devices. Kim et al. [29] developed self‐wearing adaptive garments using a fabric sheath and pressure‐driven subvines made from TPU‐coated ripstop nylon (Figure 12I). Orienting the textile reinforcement at approximately 45° to the axis reduced bending stiffness while preserving axial force transmission. Ultrasonic welding formed sealed channels, and the asymmetric surface friction of the TPU coating was oriented to promote external grip while limiting internal resistance. Because only the subvines were pressurized, the surrounding sheath remained compliant and unfurled over the body with limited squeezing. Additional subvines increased deployment force but also raised bending stiffness and the risk of jamming. This result illustrates the same material design logic as the antenna systems: functional performance is improved by separating the compliant carrier from load‐transmitting elements, but each added interface or substructure creates a new source of friction and fatigue.

These studies support a second application archetype: body‐as‐function rather than body‐as‐access. The common strategy is to decouple large deformation in the soft carrier from the smaller admissible strain of conductive or load‐transmitting elements through segmentation, textile orientation, contact‐based conductors, or separate subvines. This reduces strain incompatibility but transfers reliability to interfaces and contacts. The current evidence remains at proof‐of‐concept scale: antenna reconfiguration has been demonstrated over limited cycling, and self‐wearing garments have been evaluated under specific body geometries and deployment conditions. The unresolved material issues are puncture and leakage of the carrier, conductor or contact fatigue, weld and seam durability, coating wear, and progressive jamming as functional elements are added.

### 4.5 Comparative Discussion of Application‐Driven Material Design

The four application domains do not require different classes of vine robots so much as different allocations of the same material functions. In medical systems, low bending resistance and low interfacial force dominate, so thin membranes, coated textiles, magnetic phases, and variable‐stiffness elements are useful only if miniaturization and biological safety are preserved. In field exploration, the optimum shifts toward tear, puncture, abrasion, and environmental resistance, accepting greater wall thickness and mass. Transport tasks add pressure and medium compatibility together with local load‐bearing at the tip, whereas deployable structures make functional continuity across soft–rigid or soft–conductive interfaces a primary requirement. The practical design variable is therefore not material softness in isolation, but where compliance, reinforcement, barrier performance, friction control, and stimulus‐responsive functionality are placed along the body.

A recurring trade‐off is functionalization versus growability. Reinforcement, encapsulation, conductive layers, magnetic particles, sensors, and additional pneumatic channels can each improve task capability, yet they also introduce thickness, stiffness, areal mass, interfacial discontinuities, or friction. A recurring application‐oriented strategy is therefore spatial separation or hierarchy: a thin carrier with localized protection, a textile skeleton with a sealing coating, a working fluid that also supplies steering, segmented conductors mounted on a compliant substrate, or a soft body coupled to a mechanically stable terminal module. These architectures decouple competing functions more effectively than uniform modification of the entire body.

Experimental maturity is also uneven and should not be inferred from isolated performance numbers. Medical platforms are largely validated in phantoms or controlled models. Exploration includes several field‐relevant demonstrations, but long‐duration durability data remain sparse. Firefighting has been demonstrated at large scale in a related continuum hose system, whereas nuclear decontamination and aerial sampling remain controlled or partial task demonstrations. Electromagnetic and wearable structures show functional feasibility but limited cycle counts and application breadth. Because task metrics differ markedly across these domains, growth speed, length, or pressure alone cannot provide a meaningful cross‐application ranking.

Taken together, current applications are enabled by application‐specific material architectures rather than a universal multifunctional body. The central unresolved issue is simultaneous reliability across coupled functions: a material system that performs well during growth may fail during repeated retrieval, environmental exposure, sterilization, high‐flow transport, or electrical reconfiguration. This application‐level comparison closes the loop with Sections 2 and 3: structural materials establish the growth envelope, functional materials add controllability and task capability, and application conditions determine which property trade‐offs become dominant. The maturity of vine robotics should therefore be judged not only by whether a robot can reach a target, but by whether its material system retains the required mechanical, interfacial, and functional properties throughout the complete task cycle.

## 5 Future Directions and Summary

### 5.1 Future Directions

Despite rapid progress, vine robots remain predominantly at the stage of laboratory or controlled‐environment validation. The central challenge is no longer to demonstrate tip growth alone, but to maintain growth, steering, sensing, task execution, and recovery within the same system over a complete operating cycle. The comparisons in Sections 2, 3, 4 suggest four closely coupled directions.

First, structural materials should evolve from single‐function membranes or feedstocks toward hierarchically integrated architectures. For fluid‐driven robots, a key question is how to improve puncture, tear, abrasion, and barrier resistance without excessively increasing bending rigidity and eversion resistance. Promising candidates include multilayer films that combine thin PE or TPU pressure‐retaining layers with localized fiber reinforcement, low‐friction coatings, or damage‐tolerant elastomer skins. Self‐healing elastomers and dynamic covalent networks may provide local protection if they retain gas sealing and tolerate repeated inversion. For deposition‐based growth, low‐temperature thermoplastic blends, reversible phase‐change composites, and polymer–LMPA hybrids could improve solidification, post‐growth stiffness, or reversible softening. Their performance should be assessed through melt rheology, interlayer bonding, thermal cycling, and recoverability rather than melting temperature alone. Conducting polymers such as PEDOT: PSS, liquid‐metal conductors, ionogels, hydrogels, liquid‐crystal elastomers, dielectric elastomers, and magnetoactive composites are also attractive for sensing or local actuation. However, many are more suitable as patterned skins, embedded channels, or tip modules than as uniform pressure‐bearing bodies.

Second, manufacturability and long‐term reliability should become primary design variables rather than post hoc validation steps. Current studies commonly report growth length, pressure, or steering angle, but less often seam fatigue, coating wear, puncture tolerance, repeated eversion–retraction cycles, or environmental aging. Thin‐film systems should be evaluated at the level of resin grade, film orientation, thickness, weld geometry, and local reinforcement. Coated textiles additionally require attention to coating delamination, moisture uptake, fiber fatigue, and seam leakage. Deposition‐based robots need a different framework that includes feedstock buckling, nozzle force, interlayer fracture, cooling history, dimensional error, and thermal degradation. Qualification must also reflect the application: sterilization and biocompatibility for medicine; abrasion and weathering for exploration; chemical and thermal compatibility for transport; and electrical or contact stability for body‐as‐function devices. A meaningful benchmark should therefore cover the complete task cycle from storage and growth to interaction, retraction, and reuse.

Third, sensing and control should exploit the distinctive fact that vine robots continuously change their morphology. This complicates fixed‐body state estimation, but creates an opportunity for embodied intelligence. Distributed soft sensors, pressure and flow measurements, conductive traces, and self‐sensing materials could estimate local curvature, contact, friction, strain, leakage, and incipient buckling. Machine‐learning methods could infer unmeasured contact states, update models as body length changes, and adapt growth speed, steering, stiffness, or retraction forces in real time. The relevant question is not whether machine learning can be added to vine robots, but how learning can represent a body whose geometry and material state evolve during operation. Model‐based learning, reinforcement learning, and online system identification may be useful when contact and friction are difficult to predict. Embodied‐intelligence strategies are equally important. Passive deformation, obstacle‐aided steering, anisotropic friction, shape locking, and local compliance can transfer part of the control burden from computation to the body. A promising route is therefore co‐design of material response and control policy, so that the robot learns when to exploit environmental contact and when to actively redirect growth or change stiffness.

Fourth, future systems should be designed around application‐specific material requirements and evaluated using common cross‐platform metrics. No single material maximizes foldability, pressure capacity, stiffness, adhesion, sensing, and durability, and Section 4 shows that the dominant trade‐off changes with the task. Medical robots prioritize small diameter and low interaction force; exploration robots require damage tolerance; transport platforms require pressure and medium compatibility; and deployable structures require reliable soft–rigid or soft–conductive interfaces. Future studies should therefore report not only maximum growth length or speed, but also growth energy per unit length, tip force, path accuracy, payload‐to‐diameter ratio, and performance after relevant environmental exposure. Such reporting would allow materials to be compared through function and reliability rather than isolated performance records, and would clarify when eversion, deposition, phase transition, or hybrid growth is the most appropriate route.

### 5.2 Summary

Vine robots represent a distinct robotic paradigm in which new body length is generated or deployed near the tip while the previously formed body remains largely stationary. This architecture shifts the design emphasis toward the coupled behavior of materials, interfaces, and growth processes. Fluid‐driven eversion relies on thin films or coated textiles that balance foldability, pressure retention, and damage tolerance. Material‐deposition and phase‐transition strategies instead depend on rheology, thermal transport, interfacial bonding, and post‐growth stiffness. These routes are therefore complementary rather than directly interchangeable.

Across the functional capabilities reviewed in Section 3, current performance is enabled mainly by coordinated material–structure design rather than by a universal smart material. Steering, retraction, tip integration, variable stiffness, attachment, and branching redistribute compliance, friction, adhesion, magnetic response, or phase state in different ways, and their experimental maturity remains uneven. The applications in Section 4 reinforce the same conclusion: practical performance depends on where protection, sealing, sensing, transport, and task‐level stiffness are placed, and whether these functions remain reliable throughout the full operating cycle.

The next stage of vine robotics will therefore require more than softer or more responsive materials. It will require hierarchical, manufacturable material systems that can grow, sense, adapt, recover, and remain reliable under realistic environmental exposure. Combining such material architectures with learning‐based control and embodied intelligence may ultimately shift the field from material‐enabled growth toward material‐enabled adaptive behavior.

## Author Contributions

**Jiahao Wu**: writing – review and editing, writing – original draft, formal analysis. **Xingyun Liu**: writing – original draft, formal analysis, writing – review and editing. **Fangzhou Wang**: formal analysis, writing – review and editing. **Huili Ren**: formal analysis, writing – review and editing. **Chaojun Zhang**: formal analysis, writing – review and editing. **Ziyu Ren**: supervision, writing – review and editing, conceptualization, funding acquisition. **Zunqi Liu**: formal analysis, writing – review and editing. **Jing Li**: conceptualization, supervision, writing – original draft, writing – review and editing, funding acquisition, project administration.

## Conflicts of Interest

The authors declare no conflicts of interest.

## Data Availability

The data that support the findings of this study are available from the corresponding author upon reasonable request.
